# Thermoadaptive Supramolecular α-Cyclodextrin Crystallization-Based Hydrogels via Double Hydrophilic Block Copolymer Templating

**DOI:** 10.3390/polym10060576

**Published:** 2018-05-23

**Authors:** Tingting Li, Baris Kumru, Noah Al Nakeeb, Jochen Willersinn, Bernhard V. K. J. Schmidt

**Affiliations:** 1 Max Planck Institute of Colloids and Interfaces, 14424 Potsdam, Germany; litingting@dlut.edu.cn (T.L.); Baris.Kumru@mpikg.mpg.de (B.K.); Noah.AlNakeeb@mpikg.mpg.de (N.A.N.); jochenwillersinn@googlemail.com (J.W.); 2 State Key Laboratory of Fine Chemicals, Department of Polymer Science and Engineering, Dalian University of Technology, Dalian 116024, China

**Keywords:** double hydrophilic block copolymer, cyclodextrin, hydrogel, supramolecular chemistry

## Abstract

Supramolecular hydrogels play a prominent role in contemporary research of hydrophilic polymers. Especially, hydrogels based on α-cyclodextrin/poly(ethylene glycol) (α-CD/PEG) complexation and crystal formation are studied frequently. Here, the effect of double hydrophilic block copolymers (DHBCs) on α-CD/PEG hydrogel properties is investigated. Therefore, a novel DHBC, namely poly(*N*-vinylpyrrolidone)-*b*-poly(oligo ethylene glycol methacrylate) (PVP-*b*-POEGMA), was synthesized via a combination of reversible deactivation radical polymerization and modular conjugation methods. In the next step, hydrogel formation was studied after α-CD addition. Interestingly, DHBC-based hydrogels showed a significant response to thermal history. Heating of the gels to different temperatures led to different mechanical properties after cooling to ambient temperature, i.e., gels with mechanical properties similar to the initial gels or weak flowing gels were obtained. Thus, the hydrogels showed thermoadaptive behavior, which might be an interesting property for future applications in sensing.

## 1. Introduction

Hydrogels comprise an important class of crosslinked soft materials due to their resemblance to biological tissues with remarkable properties like swelling and elasticity [1,2]. Likewise, hydrogels hold great promises for future applications in biomedicine or biomimetic materials. Thus, hydrogels have been in the focus of research in particular regarding tissue engineering [3], cell culture scaffolds [4] or wastewater treatment [5]. Crosslinking is one of the major factors to tune hydrogel properties, in particular via the applied crosslinking chemistry and crosslinking density. In principle, there are various ways to form hydrogels via crosslinking reactions, for example via photopolymerization [6,7] or free radical polymerization as well as mechanical interlocking [8,9]. The mechanical properties of hydrogels strongly depend on crosslinking density and hydrogel architecture [10,11,12] as well as the addition of reinforcer compounds [13,14].

In recent years, supramolecular motifs were introduced in the field of hydrogels [15], e.g., cucurbiturils [16], hydrogen bonds [17], coacervation [18,19], or metal complexes [20]. One remarkable feature of supramolecular chemistry and likewise supramolecular hydrogels are self-healing and adaptivity to the environment [15,21,22,23]. Especially, cyclodextrins (CDs) have been utilized frequently in polymer science for complex macromolecular architectures [24,25] and most significantly in the formation of supramolecular hydrogels or networks [25]. Due to the supramolecular nature of the gels, intriguing properties were described, e.g., redox responsive gels [26], macroscopic self-assembly [27], shape-memory materials [28], or self-healing materials [29]. Such hydrogels can be utilized for applications including cleavable gels for cell culture [30] or hydrogel actuators [31]. In addition to hydrogels formed via simple host/guest association, hydrogels formed with crosslinks from mechanical interlocking/rotaxane formation were studied often, e.g., slide-ring gels [8,9]. A prominent class of CD-based hydrogels is formed from α-CD/poly(ethylene glycol) (PEG) rotaxanes that form crystalline domains via hydrogen bonding as crosslinking points [32,33]. These α-CD-based hydrogels feature injectability [34], biocompatibility, [35] and thermo response [36], i.e., they dissolve at higher temperatures. Recently, Loh and coworkers showed the formation of an α-CD/PEG-based hydrogel via lignin precursors [37]. A PEG side chain macromonomer, oligo ethylene glycol methacrylate (OEGMA), was utilized to form grafts on hydrophobic lignin, and finally α-CD was added to generate a supramolecular hydrogel. In another study, Li and coworkers showed the hydrogel formation between PEG-*b*-poly(3-hydroxybutyrate)-*b*-PEG and α-CD [38]. Biodegradable hydrogels were obtained that might be useful for application in the field of drug delivery.

Recently, double hydrophilic block copolymers (DHBCs) have been studied for self-assembly in aqueous solution [39,40]. The self-assembly is based on the different hydrophilicity of the individual blocks, leading to a difference in osmotic pressure and demixing of the blocks, which has been termed the hydrophilic effect [41]. In such a way, the formation of particles, vesicles or micelles was shown without utilization of external stimuli [40]. For example, our team could show the formation of DHBC vesicles from pullulan-*b*-poly(*N*-ethylacrylamide) [42]. Böker and coworkers described the formation of DHBC-based micelles from poly(2-hydroxyethyl methacrylate)-*b*-poly(2-*O*-(*N*-acetyl-β-d-glucosamine)ethyl methacrylate) [43]. Moreover, poly(*N*-vinylpyrrolidone)-*b*-poly(2-ethyl-2-oxazoline) (PVP-*b*-PEtOx) DHBC particles were described [44]. One of the main drawbacks of DHBC self-assembly is the rather low efficiency compared to amphiphilic block copolymer-based self-assembly and the low stability against dilution. Therefore, crosslinking is frequently investigated, leading to hydrogel domains. In such a way, crosslinking of a pullulan-*b*-POEGMA was performed with sodium trimetaphosphate [45]. Schubert and coworkers reported the formation of core crosslinked nanogels based on poly(2-oxazoline) DHBCs via crosslinking in organic solution [46]. Hydrogels from a double hydrophilic star block copolymer were presented by Ito and coworkers, who utilized crosslinking of a poly(acrylic acid) block via Ca^2+^ [47]. Moreover, DHBCs were utilized in the formation of mesocrystals, e.g., for minerals or metal-organic mesocrystals, showing a significant effect of DHBC addition on crystal growth [48,49]. Nevertheless, the effect of DHBCs on the formation of supramolecular hydrogels has not been in the focus of research so far.

**Scheme 1:**
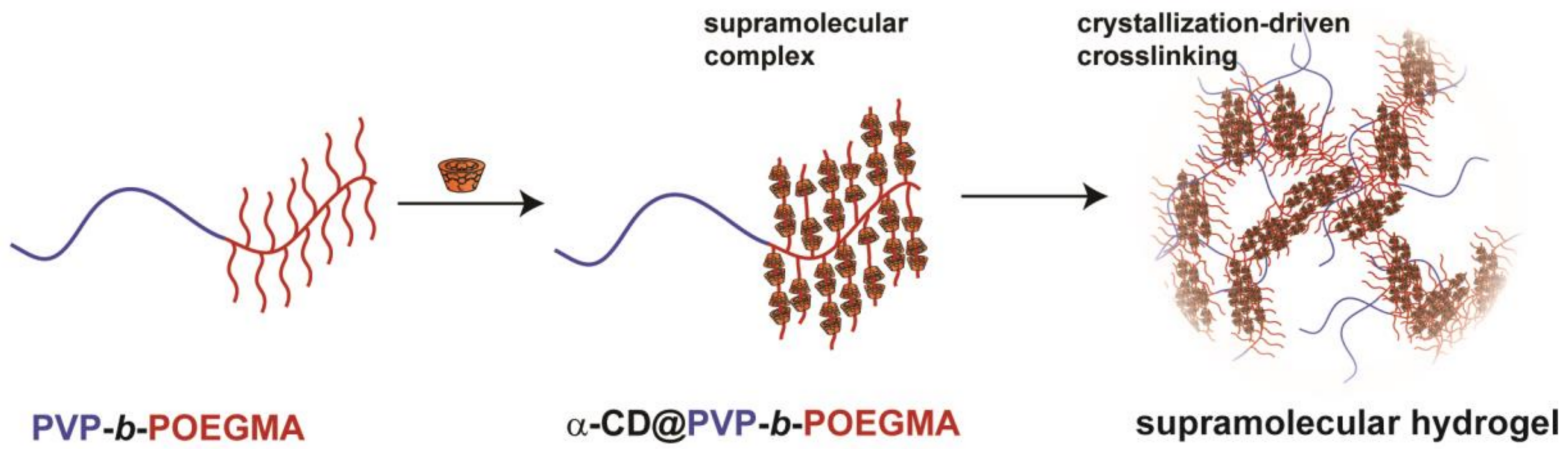
Overview of the formation of double hydrophilic block copolymer (DHBC)-mediated thermoadaptive supramolecular hydrogels.

Herein, we describe the effect of a DHBC, namely PVP-*b*-POEGMA, on the formation of α-CD-based hydrogels via supramolecular rotaxane formation followed by crystallization (Scheme 1). Soft hydrogels are obtained and the phase diagram of gel formation studied. The hydrogels show remarkable thermoadaptive features, i.e., the mechanical properties of the hydrogels depend on the thermal history. In such a way, the hydrogel can be heated to the point when sol is formed and cooled to ambient temperature, which leads to flowing gels. On the other hand, heating above the cloud point of the gels and cooling to ambient temperature leads to hydrogels with mechanical properties similar to the properties of the initial gels. The gels are characterized via rheology, powder X-ray diffraction (XRD), and (cryo) scanning electron microscopy (SEM).

## 2. Materials and Methods

### 2.1. Chemicals

α-Cyclodextrin (α-CD, 98%, Roth, Karlsruhe, Germany), ammonium chloride (99%, Roth), ascorbic acid (98%, Alfa Aesar, Karlsruhe, Germany), 2-bromopropionyl bromide (97%, Sigma Aldrich, Steinheim, Germany), *t*-butyl hydroperoxide (70% solution in water, Acros Organics, Geel, Belgium), chloromethyl polystyrene resin (2.4 mmol g^−1^, TCI), copper(I)bromide (CuBr, 99.99%, Sigma Aldrich), copper(II)sulfate (CuSO_4_, 99%, Roth), diethyl ether (ACS reagent, Sigma Aldrich), *N,N*-dimethylformamide (DMF, analytical grade, Sigma Aldrich), dimethylsulfoxide (DMSO, analytical grade, VWR Chemicals, Darmstadt, Germany), 4,4′-dinonyl-2,2′-dipyridyl (dNBipy, 97%, Sigma Aldrich), ethyl acetate (EtOAc, analytical grade, Chem Solute, Berlin, Germany), hexane (analytical grade, Fluka, Schwerte, Germany), magnesium sulfate (dried, Fisher Scientific, Schwerte, Germany), methanol (MeOH, analytical grade, Fisher Scientific), *N*-methylpyrrolidone (NMP, GC grade, Fluka), *N*,*N*,*N*′,*N*″,*N*″-pentamethyldiethylenetriamine (PMDETA, 98%, Sigma Aldrich), potassium-*O*-ethyl xanthate (98%, Alfa Aesar), propargyl alcohol (99%, Sigma Aldrich), pyridine (99% extra dry, Acros Organics), sodium azide (>99.5%, Fluka), sodium bicarbonate (>99%, Fluka), sodium sulfite (97%, Acros Organics), and triethylamine (99.5%, Sigma Aldrich) were used as received. *N*-Vinylpyrrolidone (VP, 99%, Sigma Aldrich) was dried over anhydrous magnesium sulfate and purified by distillation under reduced pressure. Oligo(ethylene glycol) methyl ether methacrylate (OEGMA, 900 g mol^−1^, Sigma Aldrich) was first dissolved in tetrahydrofuran (THF), then passed over a basic aluminum oxide column (Brockman I, Sigma Aldrich) and subsequently precipitated in cold hexane, filtered and dried under high vacuum for 24 h. Acetone (analytical grade, J.T. Baker, Schwerte, Germany) and dichloromethane (DCM, analytical grade, Acros Organics) were stored over molecular sieves (3 Å) prior to use. Millipore water was obtained from an Integra UV plus pure water system by SG Water (Hamburg, Germany). Azido functionalized PS-resin (Figure S1), prop-2-yn-1-yl 2-((ethoxycarbonothioyl)thio) propanoate (alkyne-CTA), alkyne end functionalized PVP_41k_ (Figure S2) and azide end functionalized POEGMA_22k_ (Figure S3) were prepared according to the literature (refer to the SI for details) [42,45,50]. Spectra/Por dialysis tubes with MWCO of 10,000 were purchased from Spectrum Labs (Los Angeles, CA, USA).

### 2.2. Synthesis of PVP_41k_-b-POEGMA_22k_

The conjugation reaction was performed according to the literature [51]. In a dry, argon-purged 25 mL round bottom Schlenk flask, alkyne end functionalized PVP_41k_ (0.143 g, 0.015 mmol, 1.2 eq.) was dissolved in deionized water (5.0 mL). CuSO_4_ (1.3 mg, 8.0 µmol, 0.65 eq.) and DMSO (5.0 mL) were added to the solution. Azide end functionalized POEGMA_22k_ (0.25 g, 0.0125 mmol, 1.0 eq.) and PMDETA (4.0 µL, 0.0188 mmol, 1.5 eq.) were dissolved in DMSO (2.0 mL) and added to the reaction mixture. Finally, ascorbic acid (4.4 mg, 0.025 mmol, 2.0 eq.) was added twice, once directly at the beginning of the reaction then after 24 hr. The reaction mixture was stirred at ambient temperature for 48 hr. Azido-functionalized PS-Resin (8.0 mg, 0.018 mmol) and ascorbic acid (4.4 mg, 0.025 mmol, 2.0 eq.) were added, and the reaction mixture was stirred for additional 48 hr. The resin was filtered off and the solution was dialyzed against deionized water for three days, followed by lyophilization to afford PVP_41k_-*b*-POEGMA_22k_ (0.348 g, 0.018 mmol, 88% recovery *M*_n,SEC_ = 51,000 g·mol^−1^, PMMA standard in NMP, *Ð* = 1.42) as a white powder. 

### 2.3. Exemplary Formation of Thermoadaptive PVP_41k_-b-POEGMA_22k_-Based Supramolecular Hydrogel

In a vial, PVP_41k_-*b*-POEGMA_22k_ (100.0 mg) was dissolved in Millipore water (300 mg). Subsequently, a 12 wt.% solution of α-CD in MiliQ water (2.6 mL) was added and mixed. After one minute, the solution started to turn turbid and viscous. The viscosity increased over the next 5 min until a hydrogel was obtained. Heating to 65 °C led to a viscous sol that had the character of a flowing gel after cooling to ambient temperature. Heating to 85 °C resulted in a clear solution that formed a hydrogel after cooling to ambient temperature. In subsequent experiments, the concentrations and ratios of the reactants were varied to assess the boundaries of hydrogel formation.

### 2.4. Characterization Methods

^1^H spectra were recorded at ambient temperature at 400 MHz with a Bruker Ascend400 (Billerica, MA, USA). Scanning electron microscopy (SEM) and cryogenic scanning electron microscopy (cryo SEM) was performed on a Jeol JSM 7500 F (Tokyo, Japan) equipped with an Oxford Instruments X-MAX 80 mm^2^ detector (Abingdon-on-Thames, UK) and the cryo-chamber from Gatan (Alto 2500, Munich, Germany). Size exclusion chromatography (SEC) for POEGMA and PVP was conducted in NMP with 0.05 mol L^−1^ LiBr and BSME as internal standard at 70 °C using a column system by PSS GRAM 100/1000 column (8 × 300 mm, 7 µm particle size) with a PSS GRAM precolumn (8 × 50 mm), a Shodex RI-71 detector (Munich, Germany) and a poly(methyl methacrylate) calibration with standards from PSS. Fourier transform infrared (FT-IR) spectra were acquired on a Nicolet iS 5 FT-IR spectrometer (Thermo Fisher Scientific, Schwerte, Germany). For rheological investigations hydrogels were characterized with an Anton Parr MCR 301 rheometer (Graz, Austria) equipped with a cone plate 12 (CP-12). Measurements were performed at constant angular frequency of 10 rad/s with strain range from 0.1–100% with 31 measuring points and a 0.02 mm gap. Frequency dependent measurements were performed at constant strain of 1% with a frequency range from 1–100 rad/s with 0.02 mm gap. X-ray diffraction (XRD) patterns were obtained using Bruker D8 Advance X-ray diffractometer (Billerica, MA, USA) via Cu-Kα radiation. Differential scanning calorimetry (DSC) was performed with a Netzsch DSC204 system (Selb, Germany). Glass transition temperatures (*T*_g_) were determined using the Netzsch Proteus software (Selb, Germany). Measurements were carried out under nitrogen atmosphere. Samples were first heated from ambient temperature to 300 °C, then cooled to −70 °C, then heated to 300 °C, and then cooled to −70  °C to erase thermal history. Samples were then heated to 300 °C to analyze thermal behavior. Measurements were carried out with a heating rate of 10 K min^−1^ and a holding time of 10 min in between the heating/cooling steps. The theoretical inclusion ratio (α-CD/guest ratio) was calculated according to the molecular weight of the POEGMA block, the respective degree of polymerization (DP), the molecular weight of the grafts, the DP of the grafts and the literature-known optimum ratio of α-CD/EG units of 1:2 [33].

## 3. Results and Discussion

### 3.1. Synthesis of DHBC PVP-b-POEGMA and Formation of Supramolecular Hydrogels

**Scheme 2:**
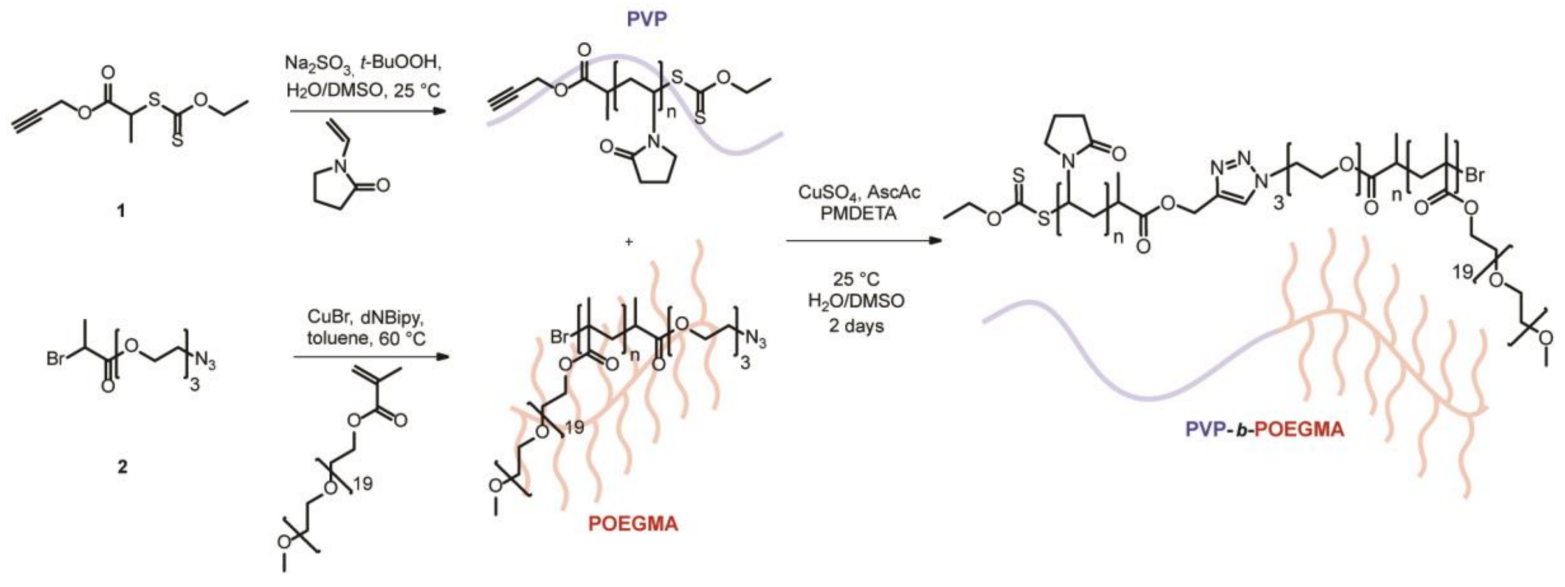
Synthesis of poly(*N*-vinylpyrrolidone)-*b*-poly(oligo ethylene glycol methacrylate) (PVP-*b*-POEGMA) via copper catalyzed azide-alkyne cycloaddition (CuAAc).

To study the effect of DHBC on α-CD-based supramolecular hydrogels, PVP-*b*-POEGMA was synthesized. Therefore, azide end-functionalized PVP was conjugated to alkyne end-functionalized POEGMA via a copper catalyzed azide-alkyne cycloaddition (CuAAc) (Scheme 2) [42]. Azide end-functionalized PVP was synthesized via reversible addition-fragmentation chain transfer polymerization of VP employing an azide-functionalized xanthate chain transfer agent (1) [44].

**Figure 1:**
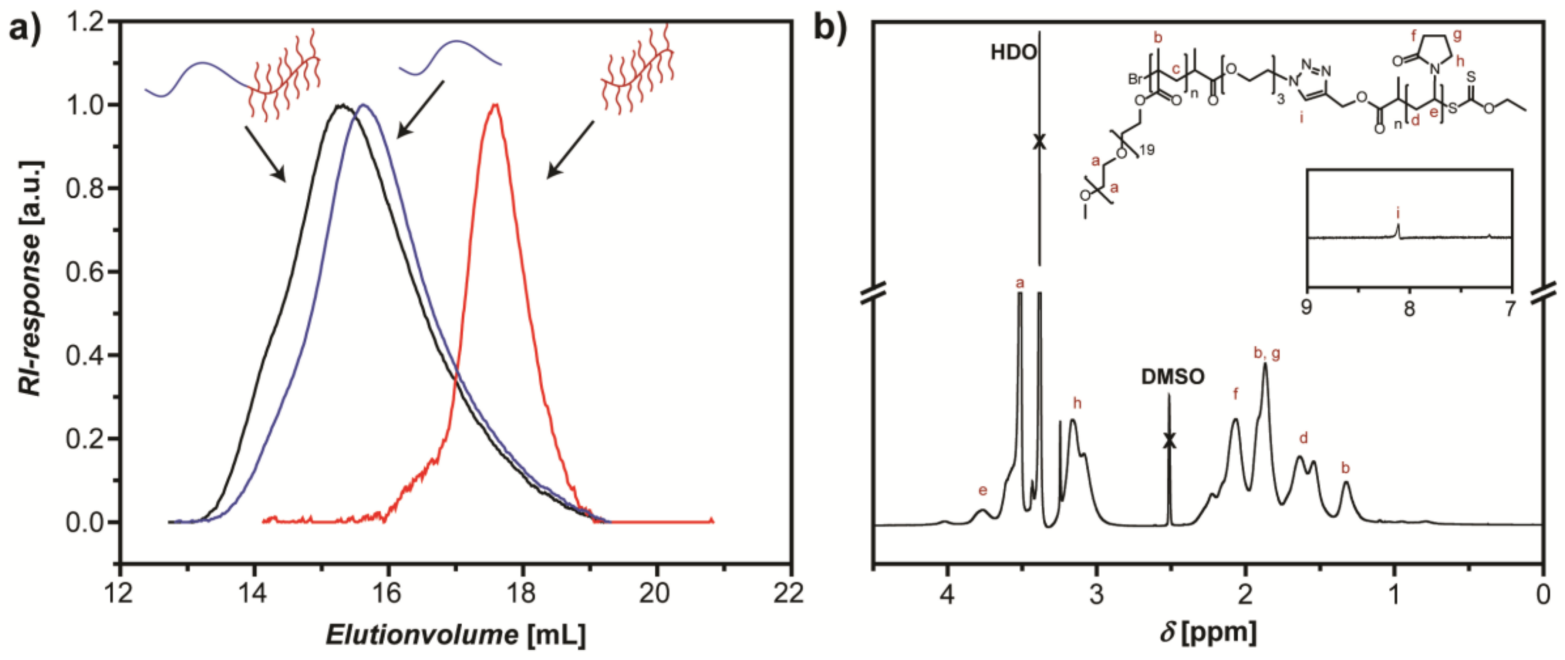
Characterization of PVP_41k_-*b*-POEGMA_22k_: (**a**) Size exclusion chromatography (SEC) in NMP at 70 °C (red trace: POEGMA_22k_; blue trace: PVP_41k_; black trace: PVP_41k_-*b*-POEGMA_22k_) and (**b**) ^1^H NMR of PVP_41k_-*b*-POEGMA_22k_ measured in DMSO-*d*_6_, inset shows the signal for the triazole proton.

For the PVP building block, a *M*_n,SEC_ of 40,600 g mol^−1^ with a *Ð* of 1.43 according to a DP of 365 was obtained. The complimentary POEGMA block was synthesized via atom transfer radical polymerization of OEGMA with a *M*_n_ of 900 g mol^−1^ and an alkyne functionalized initiator (2) [45]. A *M*_n,SEC_ of 21,700 g mol^−1^ and a *Ð* of 1.06 were achieved, which corresponds to a DP of 24. Subsequently, both building blocks were coupled via CuAAc employing the CuSO_4_/PMDETA and ascorbic acid system. The purification of the product was performed via addition of an azide functionalized resin and subsequent dialysis. The product formation could be verified via ^1^H NMR and SEC (Figure 1). ^1^H NMR shows the occurrence of both building blocks, e.g., the signal for the OEG sidechain at around 3.5 ppm and the signal for methylene protons next to the nitrogen in the VP at around 3.2 ppm. Furthermore, the signal for the triazole proton was visible (at around 8 ppm). Moreover, SEC showed a clear shift towards lower retention times, indicating successful block copolymer formation. A *M*_n,SEC_ of 51,000 g mol^−1^ with a *Ð* of 1.42 according to PMMA calibration was obtained for the block copolymer. A comparison of FT-IR spectra of the building blocks and the product supported successful coupling as the stretching band corresponding to the azide moiety (around 2100–2150 cm^−1^) was not present in the product (Figures S4 and S5).

**Figure 2:**
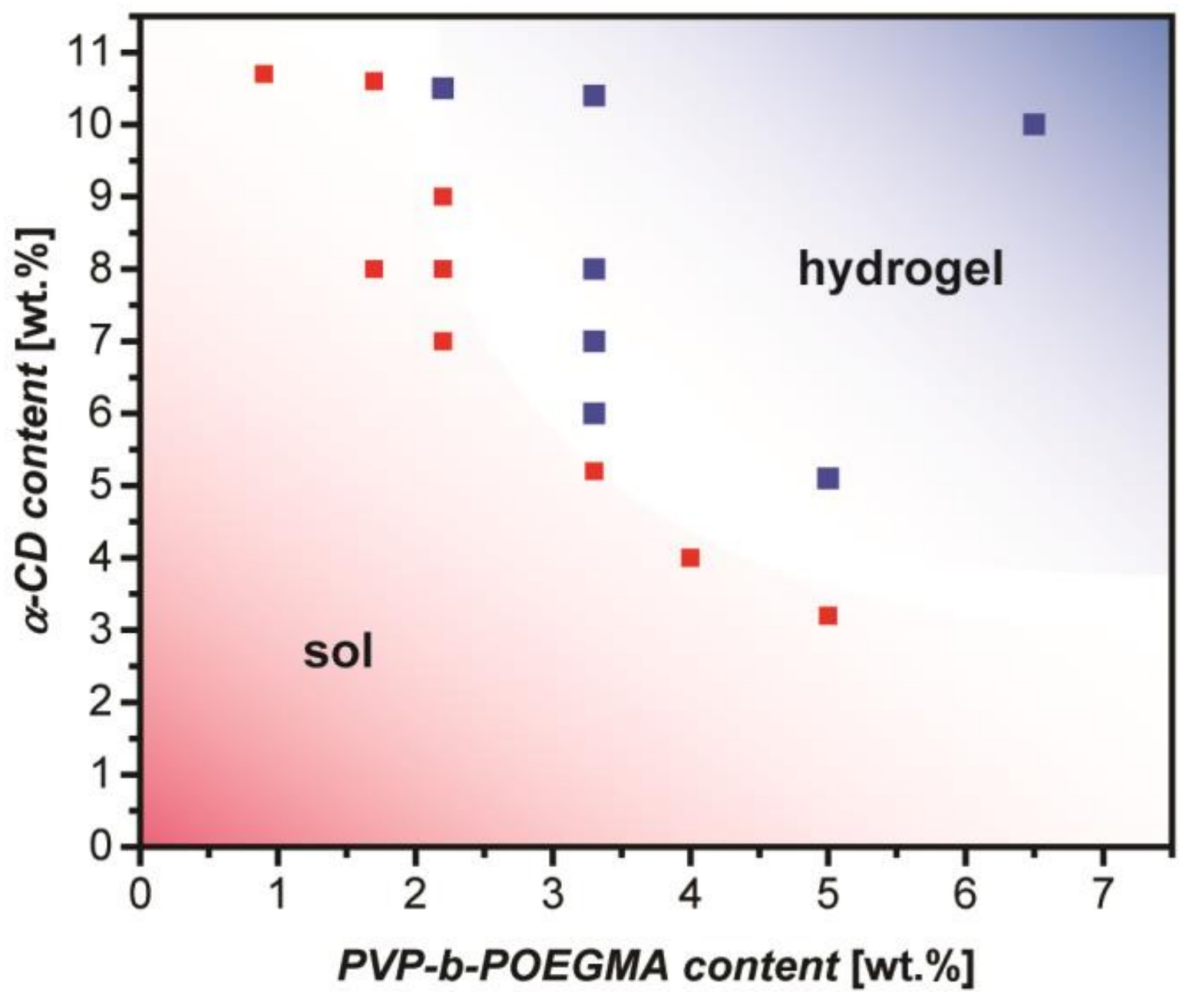
Phase diagram of PVP_41k_-*b*-POEGMA_22k_/α-CD mixtures: blue data points correspond to hydrogel and red data points correspond to sol.

In a subsequent step, the formed DHBC was combined with α-CD to form supramolecular hydrogels. Therefore, an aqueous solution of DHBC was mixed with an aqueous solution of α-CD. Depending on DHBC and α-CD concentration, hydrogel or sol formation was observed (Table 1, Figure 2).

The combination of various DHBC and α-CD concentrations allowed assembly of a phase diagram (Figure 2). From the inspection of the formed materials, three states could be observed. A clear transition between flowing gel/sol and hydrogel could be observed above 2 wt.% of DHBC as well as 4 wt.% of α-CD. Accordingly, hydrogels were formed at higher DHBC and/or α-CD concentrations. Very soft flowing gels were obtained at the boundary between the phase spaces. Thus, the formation of hydrogels could be easily tailored via the employed concentration of components. Another point that had to be considered was the inclusion ratio of guest groups and α-CD.

**Figure 3:**
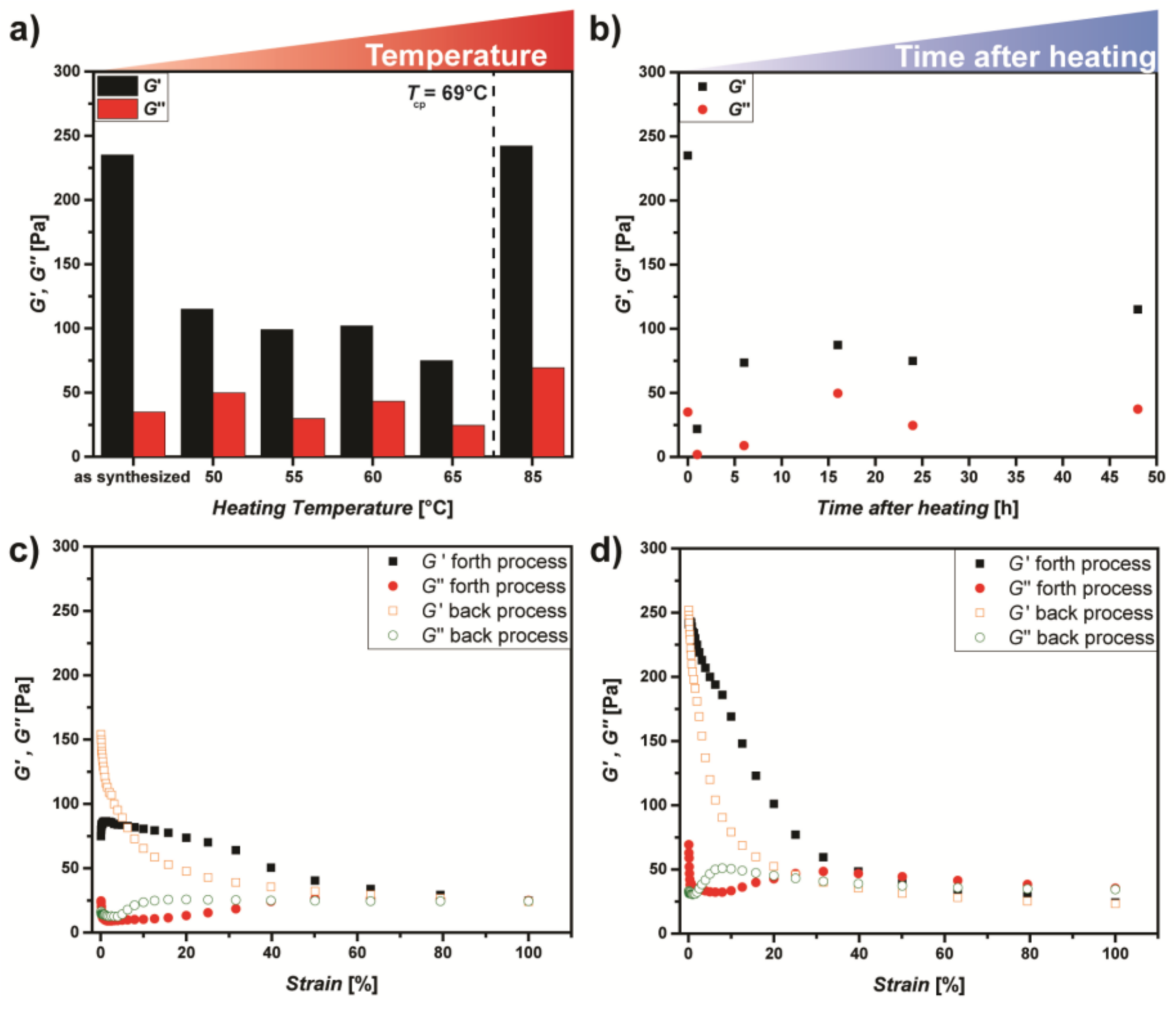
Rheological characterization of PVP_41k_-*b*-POEGMA_22k_ (3.3 wt.%)/α-CD (10.4 wt.%) hydrogel: (**a**) after heating to various temperatures, subsequent cooling to 25 °C and standing for 24 h; (**b**) after heating to 65 °C, subsequent cooling to 25 °C and standing for indicated times; (**c**) strain dependency after heating to 65 °C and cooling to 25 °C; (**d**) strain dependency after heating to 85 °C and cooling to 25 °C.

Certainly, the mechanical properties of the formed hydrogels are the most important feature. Therefore, rheology was probed subsequently. Overall, DHBC-based hydrogels were rather soft with *G*’ values below 1 kPa, e.g., 235 Pa in the case of 3.3 wt.% PVP_41k_-*b*-POEGMA_22k_/10.4 wt.% α-CD. On the other hand, reference gels from OEGMA homopolymer or the blend of PVP/POEGMA showed significantly increased *G*’ values above 1 kPa. Significant strain dependence was observed for all hydrogels (Figure 3c,d and Figures S6–S14). Especially, reference gels (pure POEGMA-based or PVP/POEGMA blend-based) that had quite a high modulus to start with, showed a significant strain dependency on moduli. For example, in the case of 1.7 wt.% POEGMA_22_/10.4 wt.% α-CD hydrogel a decrease from 3040 Pa to 195 Pa was observed when increasing the strain from 0.1% to 1.3%. Moreover, at a strain of around 1.3 %, the hydrogel was transformed into a sol, which occurred at significantly lower strains than for the DHBC-based hydrogel. Overall, the hydrogels softened up to the point, when *G*’’ exceeds *G*’ leading to a sol, which is a significant feature regarding future applications [35]. In addition, frequency dependency of *G*’ was probed for two hydrogel examples (Figures S15 and S16). A significant effect of strain frequency was observed for DHBC-based gels, for example 3.3 wt.% PVP_41k_-*b*-POEGMA_22k_/10.4 wt.% α-CD that showed an increase in modulus at low frequencies and a significant decrease at frequencies above 60 rad s^−1^. Reference hydrogels from POEGMA did not show significant effects of strain frequency. The reference sample of pure PVP block with α-CD neither formed hydrogel nor sol.

Taking the inclusion ratio into account, some other conclusions could be extracted. In general, *G*’ and *G*’’ increase with increasing inclusion ratio as long as the amount of DHBC increases, i.e., the amount of guest groups increase with respect to available α-CD. Nevertheless, in the case of increasing α-CD content with constant DHBC incorporation the hydrogel weakens, i.e., the amount of α-CD increases with respect to available guest groups. Therefore, it can be concluded that a certain threshold of α-CD incorporation is needed to strengthen the hydrogels and to obtain a strong network structure that spans through the solution.

Heating of all hydrogels as well as sols leads to transparent solutions at the cloud point *T*_cp_. The *T*_cp_ strongly depends on the α-CD concentration (Table 1), as observed via visual inspection of the solution after heating, i.e., *T*_cp_ deceases with decreased α-CD concentration. The observed *T*_cp_ are in the range of 54 to 71 °C. The clearing of the hydrogels can be explained with the complete cleavage of α-CD/PEG crystals. The hydrogels dissolve as the crystals form the crosslinks in the system [33]. Due to the thermoresponsive behavior of CD complexes, expulsion of PEG from α-CD might happen as well, which has implications for mechanical properties after cooling (see below). More interestingly the studied hydrogels feature a temperature at which the gels start to flow—the sol point *T*_sp_. *T*_sp_ is connected to the DHBC concentration, i.e., for lower concentration *T*_sp_ is decreases. A broader temperature range from 27 to 59 °C is covered by *T*_sp_. Such a behavior can be attributed to the emerging cleavage of α-CD/PEG crystals. Nevertheless, the crystals do not dissolve completely up to *T*_cp_ as the formed sols are still turbid, which is an indication of the presence of α-CD/PEG crystals.

The effect of transformation from the hydrogel to the sol and finally the solution state has significant implication on the mechanical properties. Interestingly, not only the mechanical properties at elevated temperatures are affected but also after cooling, mechanical properties depend on the previous change in physical state of the materials.

### 3.2. Thermoadaptive Properties of DHBC-Mediated Supramolecular Hydrogels

**Scheme 3:**
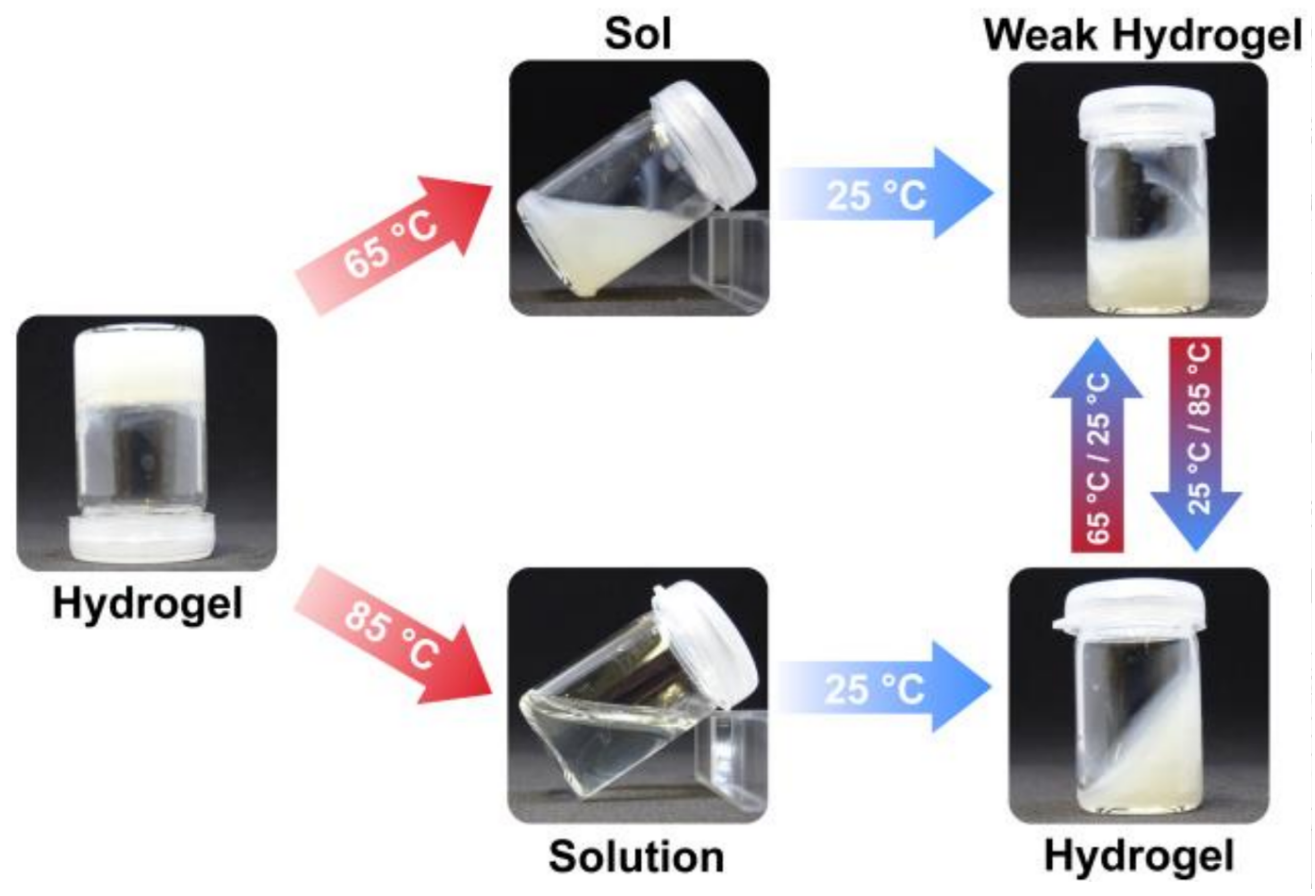
Thermoadaptive properties of DHBC-mediated supramolecular hydrogels observed on a macroscopic scale (3.3 wt.% PVP_41k_-*b*-POEGMA_22k_, 10.4 wt.% α-CD).

The thermal behavior of the formed supramolecular hydrogels leads to another striking effect (Scheme 3). As described earlier, heating of the hydrogels above *T*_sp_ led to the formation of a flowing gel. Unexpectedly, cooling to ambient temperature did not yield hydrogels with the initial properties. Significantly softer hydrogels were obtained after cooling with *G*’ around 100 Pa compared to *G*’ around 235 Pa before heat treatment. Thus, the hydrogels showed a thermoadaptive behavior being able to adapt mechanical properties depending on external thermo stimulus. Thermoadaptive behavior was observed in the case of PVP_41k_-*b*-POEGMA_22k_/α-CD 2.2 wt.%/10.5 wt.%, 3.3 wt.%/8.0 wt.% and 3.3 wt.%/10.4 wt.%. Interestingly, thermoadaptive behavior was also observed in the case of the polymer blend PVP+POEGMA/α-CD 1.7 wt.% + 1.7 wt.%/10.4 wt.%. Apparently, thermoadaptivity was strongly affected by the presence of the PVP block. Interestingly, the thermoadaptive behavior was observed only for certain inclusion ratios between 0.8 and 1.6 (guest groups/α-CD). Such a behavior is in contradiction to the effect of the inclusion ratio on the mechanical properties that showed gradual increase or decrease of mechanical strength depending on the change of the composition. Probably, the hydrogels were too weak at low inclusion ratios to show thermoadaptive behavior at all, while in the case of high inclusion ratios the hydrogels were too strong for a pronounced thermoadaptive effect.

The thermoadaptive behavior showed a weak influence of the applied temperature on the mechanical properties of the obtained flowing gels (Figure 2a and Figures S17–S19). As long as the gels were heated in the range between *T*_sp_ and *T*_cp_, similar *G*’ and *G*’’ around 100 Pa and 30 Pa were obtained respectively, which was significantly lower than the values for the initial gel. In contrast, heating above *T*_cp_ led to gels with similar strength after cooling (*G*’ 242 Pa and *G*’’ 40 Pa). Therefore, the thermoadaptivity can be reset after heating above *T*_cp_.

On the other hand, the effect of time on the mechanical properties after heating was investigated (Figure 2b and Figures S20–S23). One hour after heating and cooling, weak hydrogels were obtained (*G*’ 21.9 Pa). After six hours the gels increased in strength (*G*’ 74 Pa) and a plateau was reached. Similar *G*’ values were observed after 16, 24 and 48 h, showing the rather long-term stability of the formed flowing gels. Therefore, it is suggested that the formed flowing state of the hydrogel is kinetically trapped.

**Figure 4:**
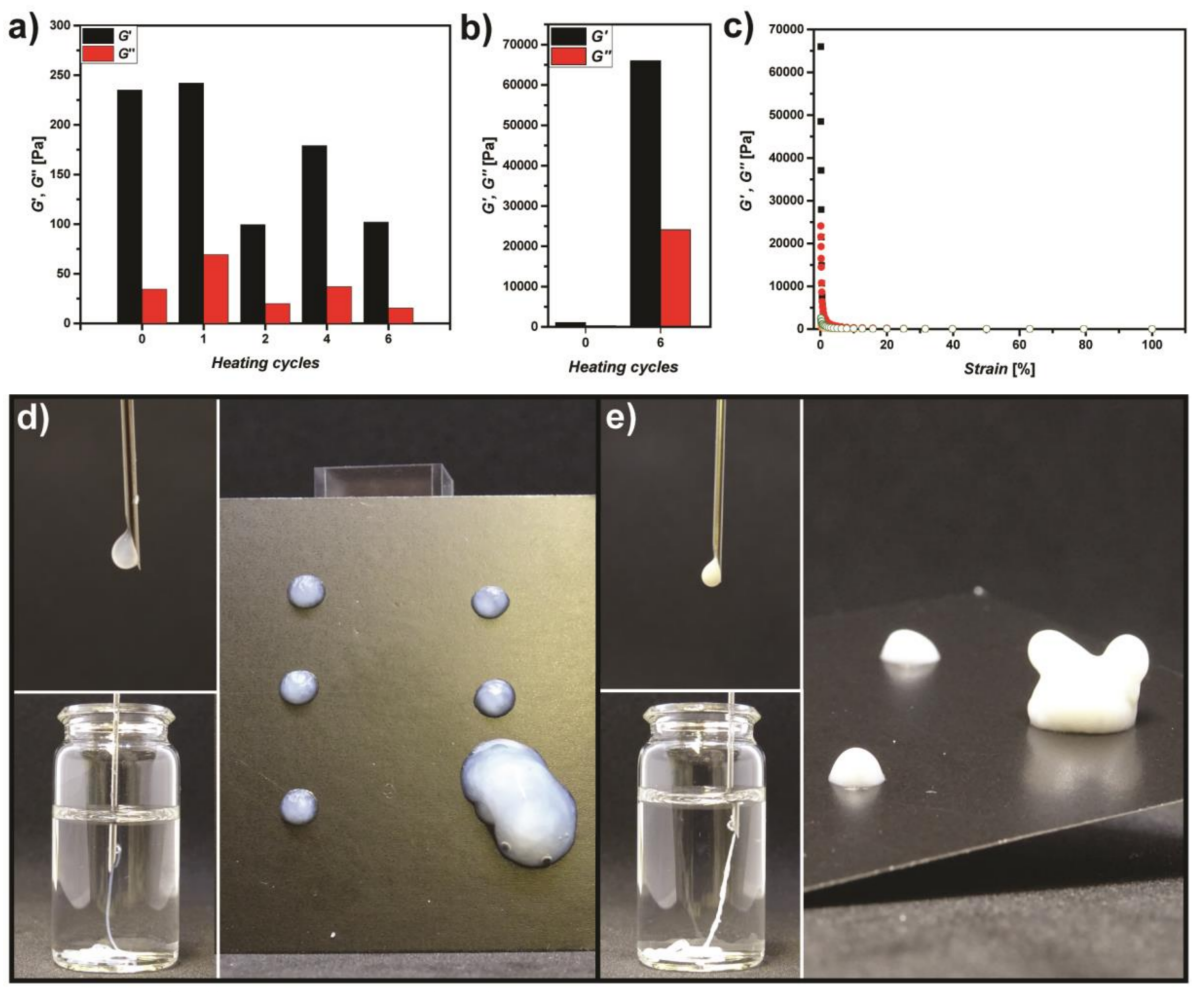
(**a**) Mechanical properties of 3.3 wt.% PVP_41k_-*b*-POEGMA_22k_/10.4 wt.% α-CD hydrogel after consecutive heating/cooling cycles between *T*_cp_ and ambient temperature, (**b**) Mechanical properties of 1.7 wt.% PVP_41k_ + 1.7 wt.% POEGMA_22_/10.4 wt.% α-CD blend hydrogel after consecutive heating/cooling cycles between *T*_cp_ and ambient temperature, (**c**) strain dependency of 1.7 wt.% PVP_41k_ + 1.7 wt.% POEGMA_22_/10.4 wt.% α-CD blend hydrogel after 6 heating/cooling cycles, (**d**) investigation of injectability for 3.3 wt.% PVP_41k_-*b*-POEGMA_22k_/10.4 wt.% α-CD hydrogel and (**e**) investigation of injectability for 1.7 wt.% PVP_41k_ + 1.7 wt.% POEGMA_22_/10.4 wt.% α-CD blend hydrogel.

It seems that the hydrogels significantly reacted on the heating above *T*_sp_ or *T*_cp_. Heating above *T*_sp_ leads to a softening, while the materials kept the gel structure with increasing strain up to 60%. Interestingly, in the back process *G*’ at 0.1% strain exceeded the initial value significantly. The strengthening effect might be due to shift of the system out of the kinetically trapped state via introduction of mechanical stress. On the other hand, heating above *T*_cp_ led to the opposite effect with similar gels after heating and a significant softening up to the formation of a sol at a strain of around 40%. Such a softening property is highly important for injectable gels as shown in Figure 4d. It can be concluded that the hydrogel thermoadaptivity does not only lead to an effect on the value of *G*’ but also on the strain dependency. Interestingly, DHBC-based hydrogels heated above *T*_sp_ or *T*_cp_ showed distinct differences in the modulus dependency on frequency (Figures S24 and S25). In the case of heating above *T*_cp_ a similar behavior to the non-temperature treated hydrogels is the case (increase at low frequencies, weak decrease above 60 rad s^−1^). For hydrogels heated above *T*_sp_, a gradual increase of modulus up to a doubled value with frequency was observed from 0 to 100 rad s^−1^. The difference of hydrogels in frequency dependent modulus measurements might be another hint towards a difference in microstructure.

Of particular interest are the control samples. The sample of POEGMA and α-CD did not show significant thermoadaptive behavior, while the blend of PVP/POEGMA and α-CD showed thermoadaptive behavior. The effect might be explained via inhibition of crystal formation due to PVP polymers in solution, similar to the recently observed effect of PVP homopolymers on metal-organic crystal formation. Accordingly, the DHBC also interacts with crystallization kinetics as shown previously [48].

Repeated heating above *T*_cp_ and cooling to ambient temperature did not lead to a strengthening effect in the case of DHBC-based hydrogels (Figure 4a and Figure S27). Heating and cooling up to six cycles did not reveal a significant difference in mechanical properties. Interestingly, a significant strengthening was observed from the blend sample PVP+POEGMA/α-CD. *G*’ values up to 66,000 Pa were obtained after six heating/cooling cycles compared to 1100 Pa before heating (Figure 4b). Probably, repeated heating above the *T*_cp_ led to a consecutive reorganization of the domains, leading to a stronger material afterwards. The effect in the blend can be attributed to the enhanced expulsion of the PVP homopolymer, while in the block, copolymer PVP is bound to the crystal-forming POEGMA block. In addition, a significant strain dependency was observed with a decrease in *G*’ to 4000 Pa at a strain of 8% (Figure 4c). At this point *G*’’ exceeds *G*’ and a sol is formed, which is a significant feature for injectability. A similar strengthening effect after heating above *T*_cp_ was observed for the POEGMA/α-CD hydrogel sample (Figure S26).

As the hydrogels show a significant strain dependency, injectability was probed. Therefore, hydrogel was introduced into a syringe and small dots of hydrogel formed after pressing through a 20 gauge needle (Figure 4d). The strain inside of the syringe was sufficient to liquefy the hydrogel and form hydrogel dots with various sizes. Moreover, the hydrogels could be injected directly into water as well, to form fiber-like gels or gel droplets. Nevertheless, the structures formed in such a way did not show long-term stability in water. Interestingly, the blend hydrogels were injectable as well, even though rather strong gels were obtained (Figure 4e), which is attributed to the significant strain dependency of the mechanical properties.

### 3.3. Microscopic Characterization of Thermoadaptive Hydrogels

A way to study the underlying crystal formation in the gelation process is powder X-ray diffraction (XRD) [33]. The crystalline nature of α-CD/PEG hydrogels is well-known and results are similar to the literature are obtained from DHBC-based hydrogels as well [38].

**Figure 5:**
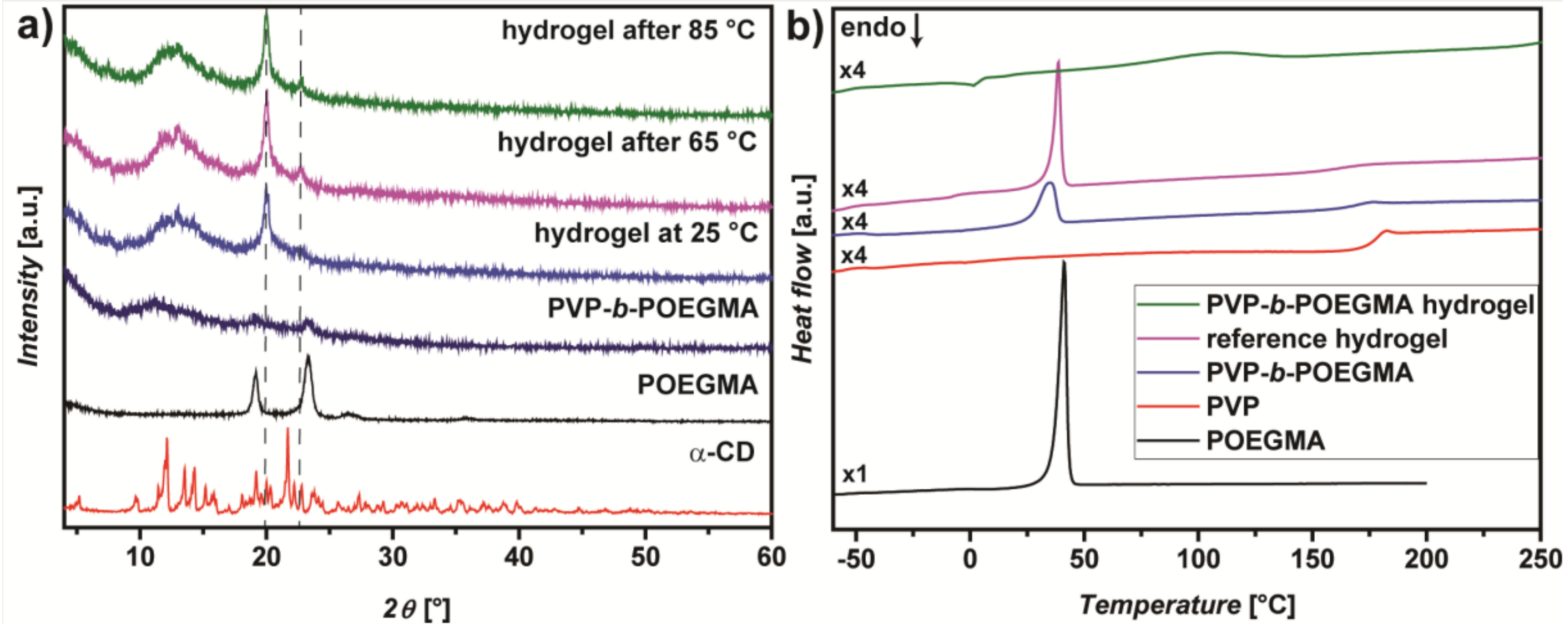
Microscopic characterization of hydrogels: (**a**) X-ray diffraction (XRD) patterns of hydrogels and individual components and (**b**) DSC traces of DHBC-based and OEGMA-based reference hydrogel.

Thus, XRD of freeze-dried DHBC-based hydrogels was studied. A sharp and strong reflex was observed at 2*θ*  =  20.0° (*d*  =  4.4 Å) as well as a weak reflex at 22.7° (*d*  = 4.0 Å) (Figure 5a and Figure S28). The reflexes were not found in the starting materials, which indicated a transformation after inclusion complex and hydrogel formation. Both reflexes were attributed to the 210 and 300 reflexes from hexagonal lattices with *a*  =  13.6 Å [52]. The strong [210] reflex is typically observed for α-CD/PEG-based hydrogels, which resembles the electron density of the α-CD core with a radius of ~5 Å. From the literature it is known that α-CD/PEG hydrogels are formed from channel-like crystallites due to the elongated PEG guest polymers. As seen from the XRD pattern of the pure POEGMA block, crystallinity was observed as well, which is due to the semi-crystallinity of PEG-based systems. In the hydrogel materials, the crystal formation of pure POEGMA domains was suppressed. Dynamic scanning calorimetry (DSC) supported the findings from XRD (Figure 5b). For the pure POEGMA_22k_ block, crystallization is observed around *T*_c_ 41.2 °C. On the other hand, PVP_41k_ has a glass transition temperature (*T*_g_) at around 182.7 °C. The DHBC PVP_41k_-*b*-POEGMA_22k_ possesses a combination of both transition temperatures, i.e., *T*_c_ of 35.1 °C and *T*_g_ of 177.5 °C. The thermal properties of freeze dried hydrogels were studied subsequently. The POEGMA-based α-CD hydrogel as a reference showed a *T*_c_ around 38.7 °C. Thus, crystallization is still possible, although complexation occurred during gel formation, which points to the fact that complexation might be not complete. Nevertheless, the crystallization peak was significantly less pronounced compared to the pure POEGMA block. In the case of freeze dried DHBC-based hydrogel, no crystallization and glass transition were observed. Thus, almost complete complexation of the POEGMA block was indicated.

**Figure 6:**
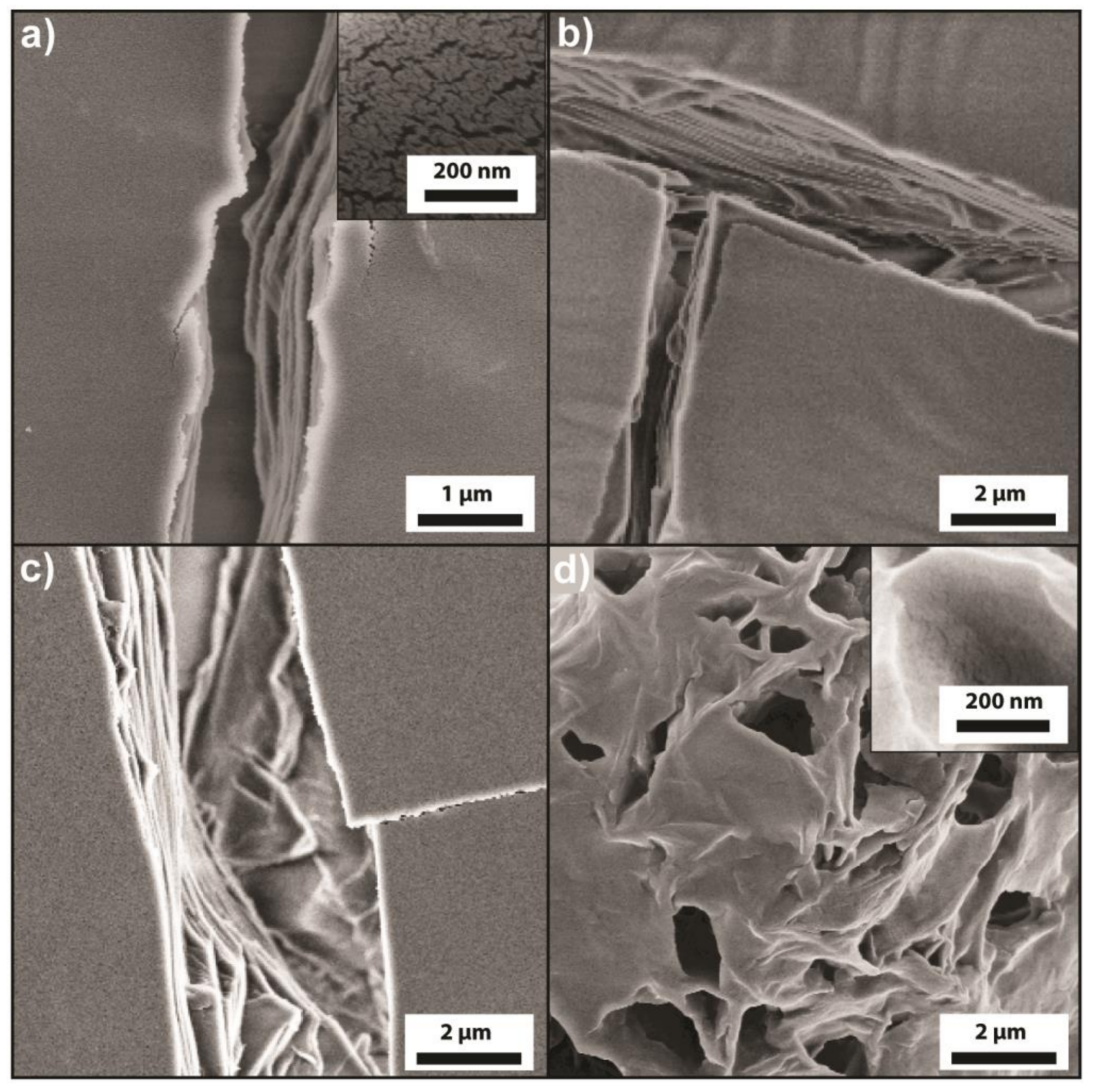
Scanning electron microscopy (SEM) imaging of freeze dried hydrogel samples: (**a**) PVP_41k_-*b*-POEGMA_22k_ (3.3 wt.%)/α-CD (10.4 wt.%) initial hydrogel, (**b**) PVP_41k_-*b*-POEGMA_22k_ (3.3 wt.%)/α-CD (10.4 wt.%) after heating to 65 °C and cooling to 25 °C, (**c**) PVP_41k_-*b*-POEGMA_22k_ (3.3 wt.%)/α-CD (10.4 wt.%) after heating to 85 °C and cooling to 25 °C and (**d**) POEGMA_22k_ (1.7 wt.%)/α-CD (10.4 wt.%) after heating to 85 °C and cooling to 25 °C.

**Figure 7:**
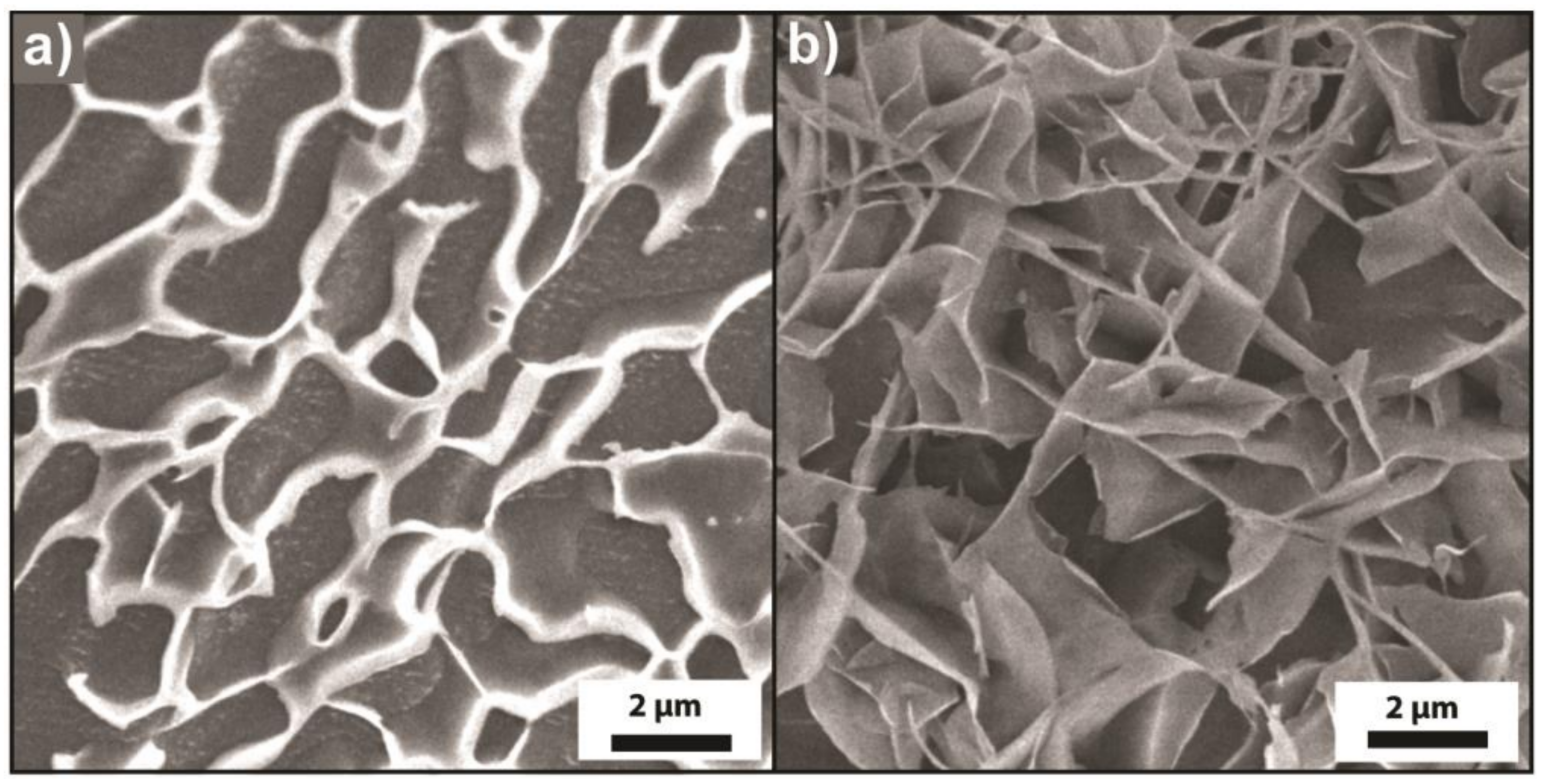
Cryo SEM imaging of hydrogel samples: (**a**) PVP_41k_-*b*-POEGMA_22k_ (3.3 wt.%)/α-CD (10.4 wt.%) and (**b**) POEGMA_22k_ (1.7 wt.%)/α-CD (10.4 wt.%).

SEM imaging of freeze dried hydrogels shows the difference between DHBC-based hydrogels and POEGMA-derived hydrogels (Figure 6). In the case of DHBC-based hydrogels layered structures are obtained, which might be due to the interplay of crystalline domains and demixing of the DHBC blocks. The layers are porous featuring pores in the sub 50 nm range (Figure 6a inset). On the other hand POEGMA-based hydrogels show a porous structure with large macropores in the micrometer range and small pores in the sub-50 nm range. Similarly layered structures were observed for trehalose-methyl cellulose-block copolymer-based hydrogel [53]. In a similar way, cryo SEM was utilized to image the hydrogel structure in a more native state (Figure 7). The porous structure of the gel can be observed clearly. In contrast to SEM images, the structures were less compact, which might be due to the suppression of drying effects. The network was formed from connected uneven wave-shaped walls. Again, the control sample of a POEGMA hydrogel showed distinct different features. A structure of interconnected flake-like plates was observed. The microscopic morphology of both hydrogels might also explain the enhanced mechanical properties of the reference compared to the DHBC-based hydrogel. In the case of the reference, a denser network of crosslinks was observed, which is a strong indication for enhanced rheological properties.

## 4. Discussion

The formation of α-CD/PEG-based hydrogels is well known, as well as the utilization of POEGMA for α-CD inclusion complex formation. In a similar way, DHBC-based supramolecular hydrogels can be formed via utilization of a POEGMA block. Compared to a POEGMA reference softer hydrogels are obtained in the case of DHBC. The hydrogels are formed via crystal formation between PEG and α-CD units that act as crosslinking points. Softer hydrogels might be formed due to the PVP blocks that hinder effective crystal formation via modulation of crystal nucleation and crystallization kinetics [48]. Thus, there is a significant effect of DHBC addition on the crystalline α-CD/PEG domain and consequently the mechanical properties of the formed gel materials are altered. The change in mechanical properties via heating above *T*_sp_ and cooling to ambient temperature can be attributed to a long-term hysteresis of mechanical properties. The hydrogels become significantly softer compared to the initial state, similar to other studies where CD complexes were utilized to modify the thermal properties of polymers with a time-delay [54,55].

The addressability of the two different mechanical states via heating above *T*_sp_ and *T*_cp_ might be due to the crystalline substructure of the hydrogel. Heating above *T*_cp_ leads to dissolution of the microcrystals and cooling to a substructure that is similar to the initial structure. On the other hand, heating above *T*_sp_ does not break the microcrystals completely. Upon cooling, the formation of a crystal structure similar to the initial microstructure might be kinetically hindered due to the PVP block that forms a steric barrier.

It seems that the hydrogels significantly react on the heating above *T*_sp_ or *T*_cp_. Heating above *T*_sp_ leads to a softening, while the materials keep the gel structure with increasing strain. On the other hand, heating above *T*_cp_ leads to the opposite effect with stronger gels after heating, and a significant softening up to the formation of a sol with increasing strain. Such a softening property is highly important for injectable gels as shown in Figure 4c,d. Moreover, the hydrogel thermoadaptivity does not only lead to an effect on the value of *G*’ but also on the strain dependency.

The crystalline sub-structure of the hydrogels could be verified via XRD, and is in-line with the literature [33]. Nevertheless, only minor differences between the different thermal treatments were observed. Moreover, DSC confirms the inclusion complex formation that leads to supramolecular gelation and the dangling PVP chains. From SEM imaging, the porous structure of the hydrogels can be verified. Interestingly, both cryo SEM and SEM show distinct differences in the pore structure between DHBC-based hydrogel and POEGMA-based hydrogel. Thus, the differences in mechanical properties can be correlated with the microstructure of the hydrogels, e.g., loose pore structure in the DHBC-case and a denser interconnected structure in the POEGMA-case.

## 5. Conclusions

The utilization of DHBC for the formation of supramolecular α-CD rotaxane host/guest hydrogel has a significant effect on the hydrogel properties. Soft hydrogels are obtained that show unprecedented thermal properties. Interestingly, the mechanical properties of the hydrogels depend on the thermal history of the material, which is probably due to the DHBC-mediated modulation of crystallization. Heating of the DHBC-based hydrogel above *T*_sp_ and cooling to ambient temperature leads to weak flowing gels. On the contrary, heating above *T*_cp_ and cooling to ambient temperature leads to gels with properties similar to the initial hydrogels. Thus, the DHBC-based hydrogels show thermoadaptive behavior. Moreover, the hydrogels feature shear thinning behavior, which leads to injectable gels. Overall, DHBC-based α-CD hydrogels show a promising platform for future developments in supramolecular hydrogels. Especially, thermoadaptive properties might be an interesting feature for future applications in sensing.

## Figures and Tables

**Table 1:** Rheological properties and transition temperatures of DHBC-based supramolecular hydrogels.

Polymer	Polymer Content [wt.%]	α-CD Content [wt.%]	Guest/α-CD Ratio	State ^1^	*G*’ [Pa] ^2^	*G*’’ [Pa] ^2^	*T*_sp_ [°C] ^1^	*T*_cp_ [°C] ^1^	Thermo-Adaptive ^1^
PVP_41k_-*b*-POEGMA_22k_	0.9	10.7	0.33	sol	26	28	-	64	-
PVP_41k_-*b*-POEGMA_22k_	1.7	10.6	0.62	weak gel	50	36	-	65	-
PVP_41k_-*b*-POEGMA_22k_	2.2	10.5	0.81	gel	60	22	27	68	x
PVP_41k_-*b*-POEGMA_22k_	3.3	10.4	1.22	gel	235	35	45	69	x
PVP_41k_-*b*-POEGMA_22k_	6.5	10.0	2.51	gel	842	170	59	71	-
PVP_41k_-*b*-POEGMA_22k_	3.3	5.2	2.45	flowing gel	57	32	-	54	-
PVP_41k_-*b*-POEGMA_22k_	3.3	8.0	1.59	gel	83	54	31	60	x
PVP_41k_ + POEGMA_22k_	1.7 + 1.7	10.4	1.26	gel	1110	253	45	71	x
POEGMA_22k_	1.7	10.4	1.80	gel	3040	964	55	72	-
PVP_41k_	1.7	10.4	-	solution	-	-	-	-	-

^1^ Obtained via macroscopic inspection, ^2^ obtained via rheology at 0.1% strain after gel formation and standing for 24 h without temperature treatment.

## Supplementary Materials

The following supplementary materials are available online at http://www.mdpi.com/2073-4360/10/6/576/s1.
