# Supplementary material for: Thermoadaptive Supramolecular α-Cyclodextrin Crystallization-Based Hydrogels via Double Hydrophilic Block Copolymer Templating
Source: Polymers (Basel). 2018 May 23;10(6):576. doi: 10.3390/polym10060576 (PMC6404023; doi:10.3390/polym10060576)
Supplement: Supplementary file 1 [file polymers-10-00576-s001.pdf]

Supporting Information

Thermoadaptive Supramolecular α-Cyclodextrin Crystallization-based Hydrogels via Double Hydrophilic Block Copolymer Templating

Tingting Li,[†,‡] *Baris Kumru,*[†] *Noah Al Nakeeb,*[†] *Jochen Willersinn,*[†] *Bernhard V.K.J. Schmidt*[†*]

[†] Max-Planck Institute of Colloids and Interfaces; Department of Colloid Chemistry, Am Mühlenberg 1, 14476 Potsdam, Germany

[‡] State Key Laboratory of Fine Chemicals, Department of Polymer Science and Engineering, Dalian University of Technology, Dalian, 116024, China

bernhard.schmidt@mpikg.mpg.de

Prop-2-yn-1-yl 2-bromopropanoate[1]

In a dry, argon purged 250 mL Schlenk tube, propargyl alcohol (3.94 g, 4.1 mL, 70.2 mmol, 1.0 eq.) and triethylamine (9.95 g, 13.6 mL, 98.3 mmol, 1.4 eq.) were dissolved in dry THF (150 mL). The reaction mixture was cooled to 0 °C and 2-bromopropionyl bromide (18.19 g, 8.83 mL, 84.24 mmol, 1.2 eq.) was added slowly dropwise to the reaction mixture. The reaction mixture was allowed to warm to ambient temperature and stirred for 5 hours at ambient temperature. The formed salt was filtered off and the organic phase was subsequently washed with 2 M HCl solution (3 × 10 mL), deionized water (3 x 30 mL), saturated $NaHCO_3$ solution (3 x 10 mL), and deionized water (3 × 30 mL). The organic phase was dried over anhydrous $MgSO_4$ and the solvent was removed under reduced pressure. The crude product was purified via vacuum distillation (98 °C, 37 mbar) to afford prop-2-yn-1-yl 2-bromopropanoate (8.0 g, 42.11 mmol, 60% yield) as slightly yellow liquid.

^{1}H NMR (400 MHz, $CDCl_3$ δ:) 4.76 (dd, ^1J = 3.8 Hz, ^4J = 2.5 Hz 2H, CH_2), 4.40 (q, ^3J = 6.9 Hz, 1H, *CH*), 2.52 (t, ^4J = 2.5 Hz, 1H, *alkyne-H*), 1.84 (d, ^3J = 6.9 Hz, 3H, CH_3).

Prop-2-yn-1-yl 2-((ethoxycarbonothioyl)thio) propanoate (**1**)[1]

In a dry, argon purged 250 mL Schlenk flask, prop-2-yn-1-yl 2-bromopropanoate (3.0 g, 15.79 mmol, 1.0 eq.) was dissolved in dry THF (200 mL). Potassium *O*-ethyl xanthate (25.78 g, 157.93 mmol, 10.0 eq.) was added portion wise to the solution under argon flow. The reaction mixture was stirred over night at ambient temperature. The formed salt and the excess of potassium *O*-ethyl xanthate was filtered off and the organic phase was washed with deionized water (4 × 75 mL) and dried over anhydrous $MgSO_4$. The solvent was evaporated to afford prop-2-yn-1-yl 2-((ethoxycarbonothioyl)thio) propanoate (3.145 g, 13.55 mmol, 86% recovery) as yellow oil.

^{1}H NMR (400 MHz, $CDCl_3$ δ:) 4.74 (d, ^4J = 2.5 Hz 2H, *CH_2*), 4.61 (q, ^3J = 7.1 Hz, 2H, *CH_2O*), 4.41 (q, ^3J = 7.4 Hz, 1H, *CH*), 2.52 (t, ^4J = 2.5 Hz, 1H, *alkyne-H*), 1.58 (d, ^3J = 7.4 Hz, 3H, *CH_3*) 1.42 (t, ^3J = 7.1 Hz, 3H, *CH_3*).

Synthesis of 2-(2-(2-azidoethoxy)ethoxy)ethyl 2-bromopropanoate (**2**)[2]

A solution of 2-bromopropionyl bromide (8.9 mL, 85.5 mmol) in anhydrous THF (100 mL) was added to a solution of triethylamine (11.9 mL, 85.5 mmol) and 2-(2-(2-azidoethyoxy)ethoxy)ethanol[3] (10.00 g, 57 mmol) in anhydrous THF (180 mL) at 0 °C. The solution was stirred at 0 °C for 1 h then left stirring overnight at ambient temperature. Afterwards methanol (30 mL) was added to react with the excess of 2-bromopropionyl bromide. The mixture was filtered and concentrated under vacuum. The residue was dissolved in dichloromethane (30 mL), washed three times with saturated ammonium chloride solution (100 mL) and twice with water (100 mL). Afterwards the solution was dried over magnesium sulfate and concentrated. The residue was purified via column chromatography on silica gel with hexane–ethyl acetate (10:1). Yield 25%.^{1}H-NMR (400 MHz, $CDCl_3$) δ (ppm): 4.42 (qd, J = 6.9, 2.9 Hz, 1H), 4.38 – 4.19 (m, 2H), 3.87 – 3.58 (m, 8H), 3.47 – 3.33 (m, 2H), 1.92 – 1.77 (m, 3H). ^{13}C-NMR (100 MHz, $CDCl_3$) δ (ppm): 40.0, 50.6, 61.6, 65.0, 68.8, 70.1, 70.7, 72.4, 170.3.

Synthesis of azidomethyl polystyrene resin[4]

In a dry, argon purged 100 mL round bottom Schlenk tube chloromethyl polystyrene resin (10.0 g, 24.0 mmol, 1.0 eq.) was dissolved in dry DMSO (50 mL). Sodium iodide (10.8 g, 72.0 mmol, 3.0 eq.) and sodium azide (15.6 g, 240.0 mmol, 10.0 eq.) were added and the reaction mixture was moderately stirred for 48 hours at 80 °C. The afforded resin was filtered

over a glass frit (pore size 3) and alternately washed with DCM (6× 30 mL) and MeOH (6 × 30 mL). The purified resin was finally washed with diethyl ether (30 mL) and dried under vacuum to afford azidomethyl polystyrene resin (9.25 g, 22.2 mmol, 93% recovery) as a white solid. FT-IR ($\tilde{\nu}$ cm^{-1}): 3120, 2855, 2089 (N_3), 1509, 1450, 750, 697.

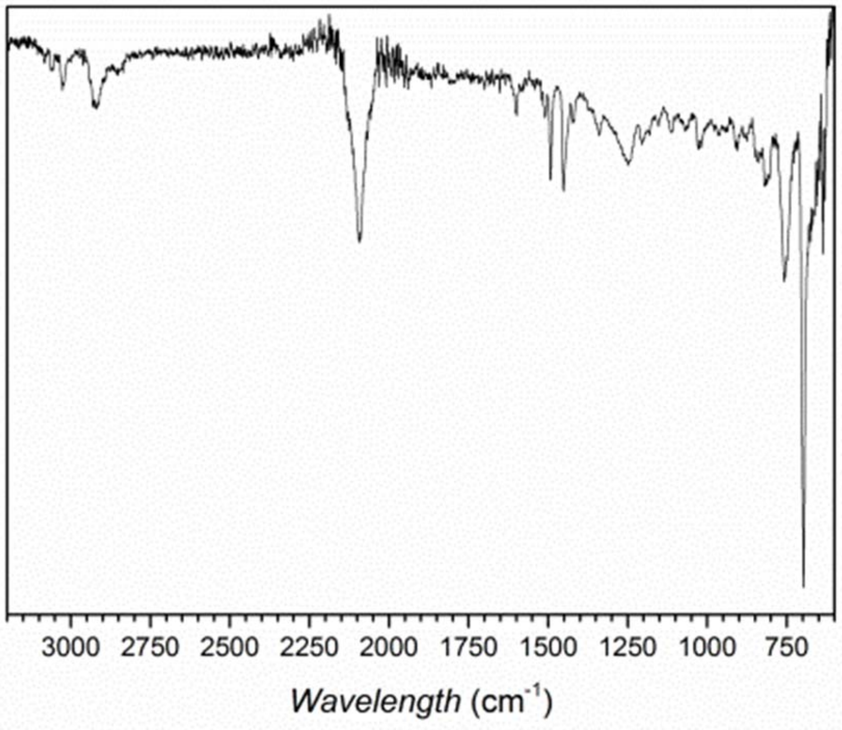

Figure S1. FT-IR spectrum of azidomethyl polystyrene resin recorded at 25 °C.

Synthesis of PVP$_{41k}$

In a dry argon, purged 25 mL Schlenk tube, **1** (0.035 g, 0.15 mmol, 1.0 eq.) was dissolved in deionized water (3.36 mL). *N*-vinylpyrrolidone (7.5 g, 67.5 mmol, 450 eq.) and *t*-BuOOH solution (4.0 mg of 70 wt.% solution, 0.068 mmol, 0.45 eq.) were added to the solution. The mixture was frozen in liquid nitrogen and sodium sulfite (3.8 mg, 0.03 mmol, 0.2 eq.) was added.

The flask was degassed via three freeze-pump-thaw cycles and immersed in an oil bath at 25 °C. After 3 h, the polymerization was quenched with liquid N_2 and exposed to air. Water was removed via reduced pressure and the crude polymer was dissolved in a small amount of MeOH. The mixture was precipitated twice into cold diethyl ether to afford alkyne terminated PVP (PVP-alkyne) as a white powder. (Yield: 3.06 g, 66%, 0.11 mmol, $M_{n,SEC}$ = 40600 g·mol^{-1} (PMMA equivalents in NMP), $Đ$ = 1.43).

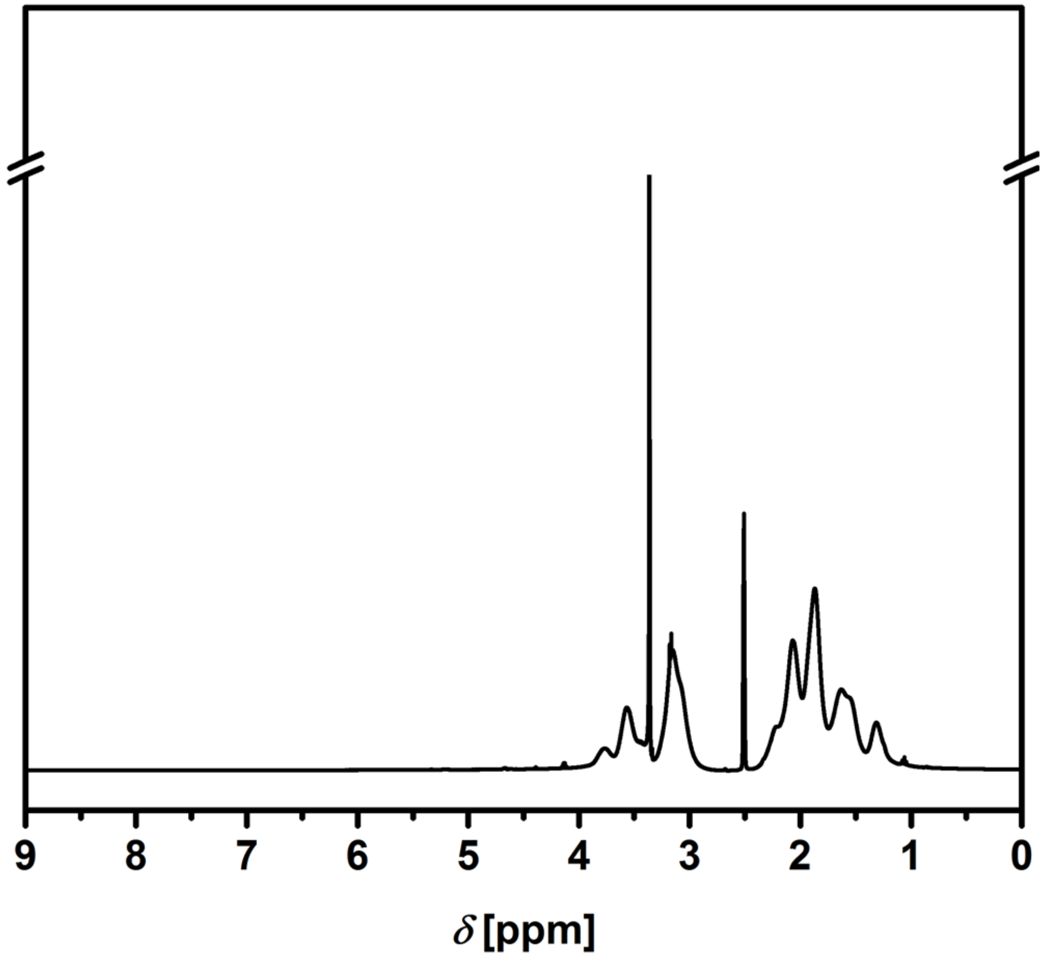

Figure S2. ^{1}H NMR spectrum of PVP_{41k} recorded at 400°MHz in DMSO-d_6.

Synthesis of POEGMA$_{22k}$

A stirring bar, **2** (62.0 mg, 0.2 mmol, 1 eq), dNbpy (165.0 mg, 0.4 mmol, 2 eq) destabilized OEGMA (3.8 g, 4 mmol, 20 eq) and toluene (7.6 mL) were placed in a Schlenk tube. The tube was first sealed with a septum then the mixture was degassed via three freeze, pump and thaw cycles followed by a last freeze and purging with Ar. While purging with Argon, CuBr (28.0 mg, 0.2 mmol, 1 eq) was added to the frozen mixture and the Schlenk tube was purged with Ar until the frozen solution thawed and the solution color changed to brown indicating the formation of the copper-complex. Subsequently, the reaction mixture was placed in an oil bath and stirred at 60 °C for 11 hours. The reaction was cooled to ambient temperature and the reaction was stopped by removing the septum allowing oxygen to enter the mixture. The solution was then concentrated and a NMR-sample was taken to determine the conversion. The residue was dissolved in THF and passed through a short column filled with neutral aluminiumoxide. The resulting solution was concentrated and the polymers precipitated in cold hexane. Subsequently the solution was filtered and dried under high vacuum. Since POEGMA was used in the following coupling reaction with PVP, no dialysis was applied at this step to remove the remaining monomer. (Yield: 1.98 g, 65%, 0.13 mmol, $M_{n,SEC}$ = 21700 g·mol^{-1} (PMMA equivalents in NMP), $Đ$ = 1.06).

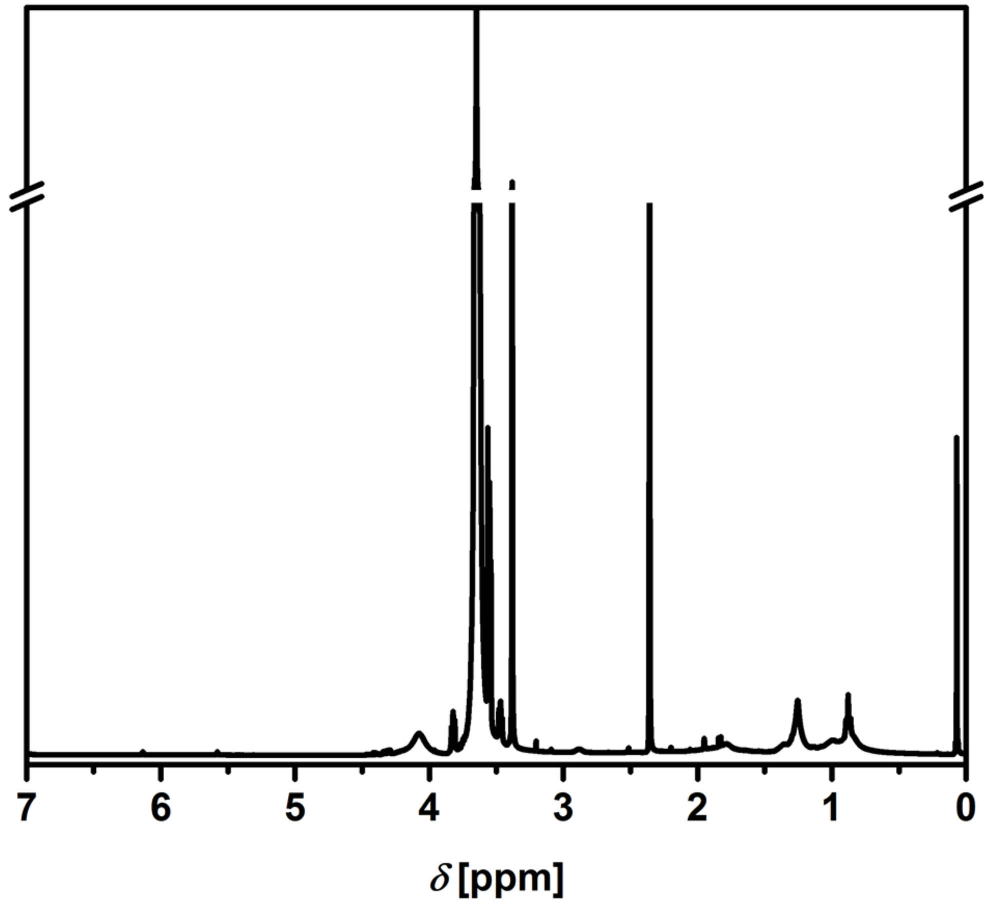

Figure S3. ^{1}H NMR spectrum of $POEGMA_{22k}$ recorded at 400°MHz in DMSO-d_6.

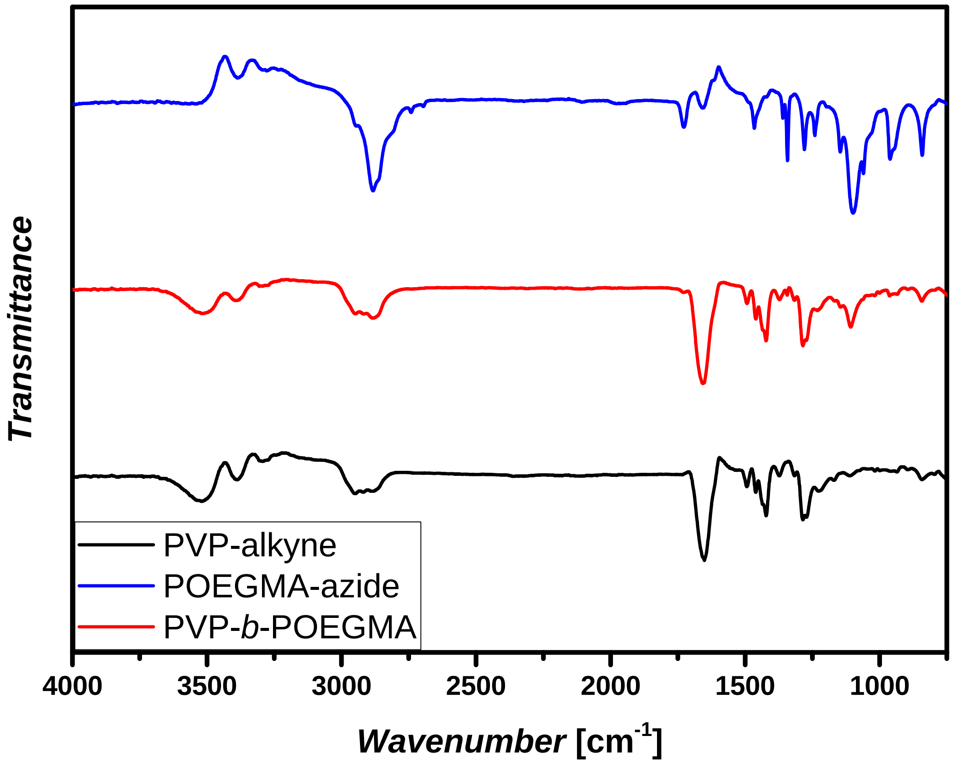

Figure S4. FT-IR spectra of $POEGMA_{22k}$, PVP_{41k} and PVP_{41k}-*b*-$POEGMA_{22k}$ recorded at 25 °C.

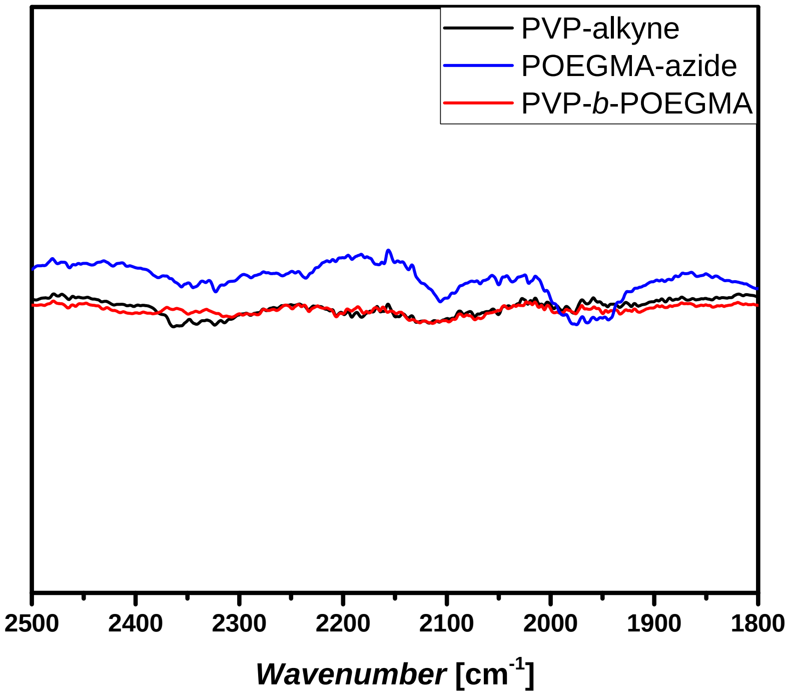

Figure S5. Magnification of FT-IR spectra of POEGMA$_{22k}$, PVP$_{41k}$ and PVP$_{41k}$-*b*-POEGMA$_{22k}$ recorded at 25 °C.

Rheological characterization

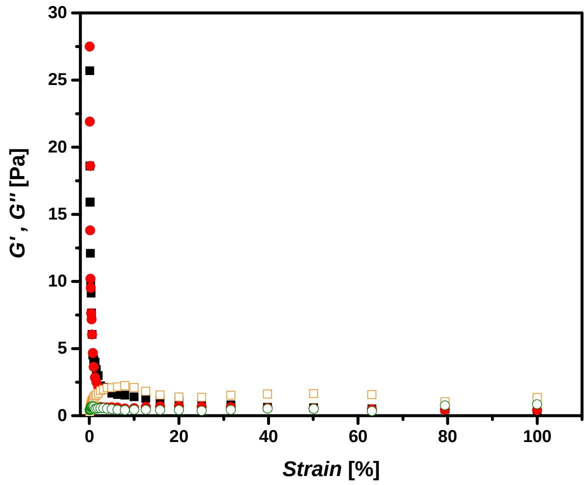

Figure S6. PVP$_{41k}$-*b*-POEGMA$_{22k}$ 0.9 wt.%/α-CD 10.7 wt.%: Comparison of storage (*G'*, black and orange) and loss modulus (*G''*, red and green) values against strain, back (open) and forth (filled) process.

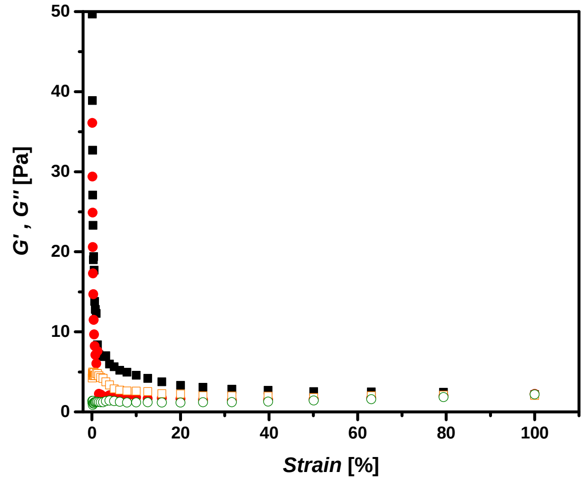

Figure S7. PVP$_{41k}$-*b*-POEGMA$_{22k}$ 1.7 wt.%/α-CD 10.6 wt.%: Comparison of storage (*G'*, black and orange) and loss modulus (*G''*, red and green) values against strain, back (open) and forth (filled) process.

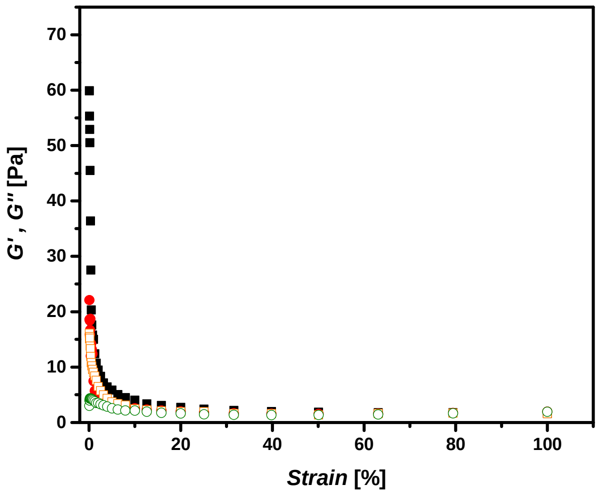

Figure S8. PVP$_{41k}$-*b*-POEGMA$_{22k}$ 2.2 wt.%/α-CD 10.5 wt.%: Comparison of storage (*G'*, black and orange) and loss modulus (*G''*, red and green) values against strain, back (open) and forth (filled) process.

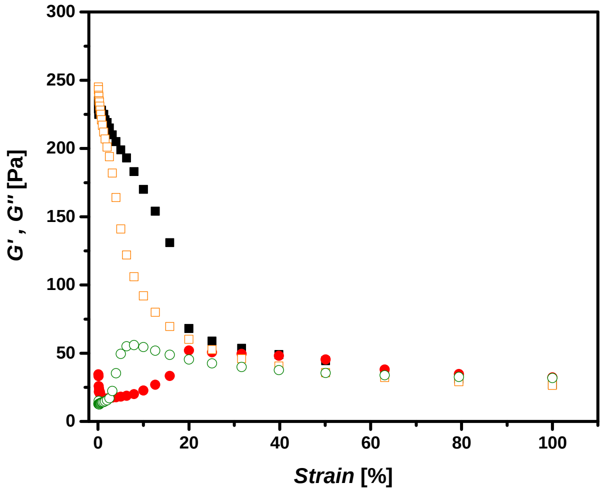

Figure S9. PVP$_{41k}$-*b*-POEGMA$_{22k}$ 3.3 wt.%/α-CD 10.4 wt.%: Comparison of storage (*G'*, black and orange) and loss modulus (*G''*, red and green) values against strain, back (open) and forth (filled) process.

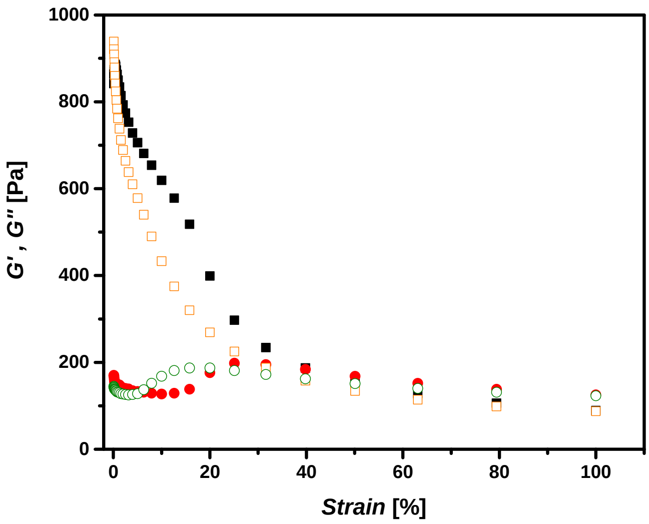

Figure S10. PVP$_{41k}$-*b*-POEGMA$_{22k}$ 6.5 wt.%/α-CD 10.0 wt.%: Comparison of storage (*G'*, black and orange) and loss modulus (*G''*, red and green) values against strain, back (open) and forth (filled) process.

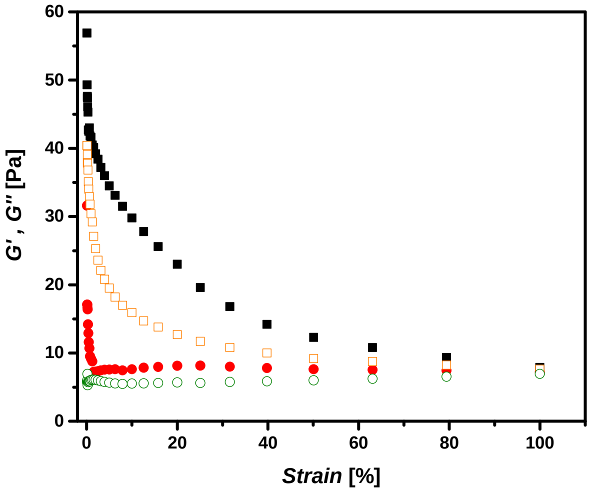

Figure S11. PVP$_{41k}$-*b*-POEGMA$_{22k}$ 3.3 wt.%/α-CD 5.2 wt.%: Comparison of storage (*G'*, black and orange) and loss modulus (*G''*, red and green) values against strain, back (open) and forth (filled) process.

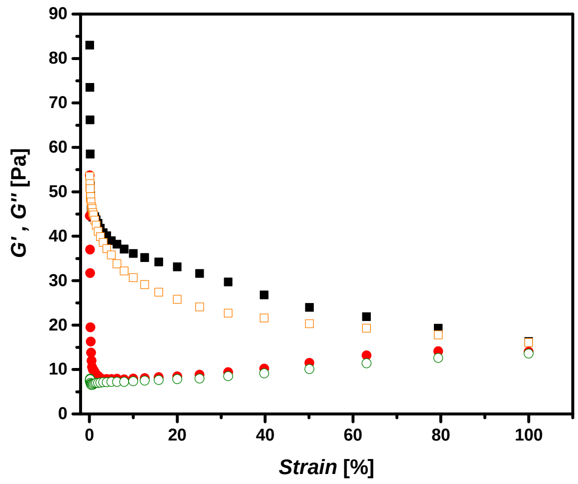

Figure S12. PVP$_{41k}$-*b*-POEGMA$_{22k}$ 3.3 wt.%/α-CD 8.0 wt.%: Comparison of storage (*G'*, black and orange) and loss modulus (*G''*, red and green) values against strain, back (open) and forth (filled) process.

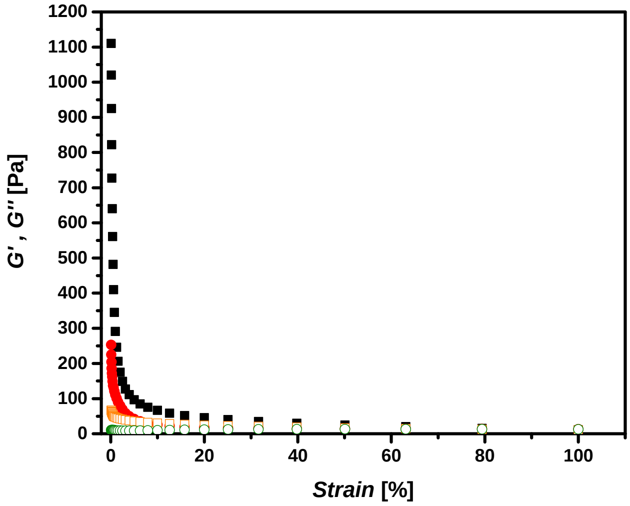

Figure S13 PVP$_{41k}$+POEGMA$_{22k}$ blend 1.7+1.7 wt.%/α-CD 10.4 wt.%: Comparison of storage (*G'*, black and orange) and loss modulus (*G''*, red and green) values against strain, back (open) and forth (filled) process.

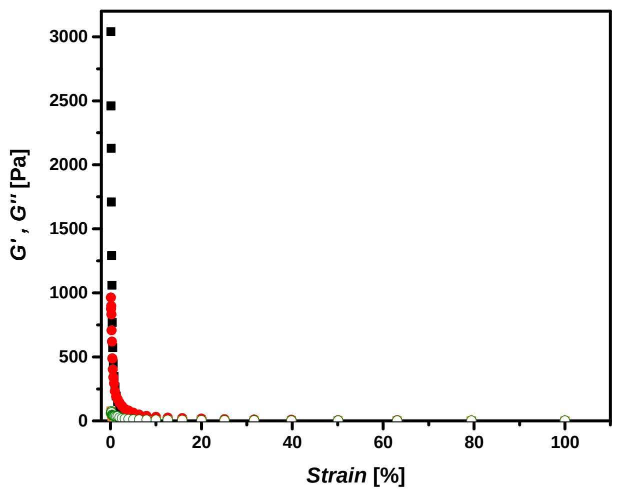

Figure S14 POEGMA$_{22k}$ 1.7 wt.%/α-CD 10.4 wt.%: Comparison of storage (*G'*, black and orange) and loss modulus (*G''*, red and green) values against strain, back (open) and forth (filled) process.

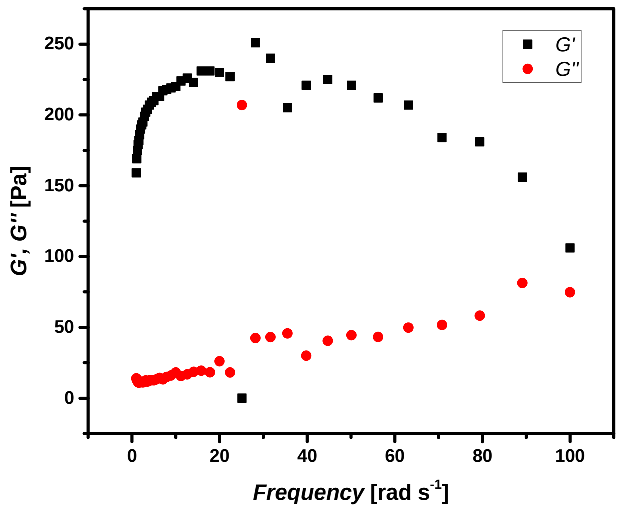

Figure S15. PVP$_{41k}$-*b*-POEGMA$_{22k}$ 3.3 wt.%/α-CD 10.4 wt.%: Comparison of storage (*G'*, black) and loss modulus (*G''*, red) values against frequency.

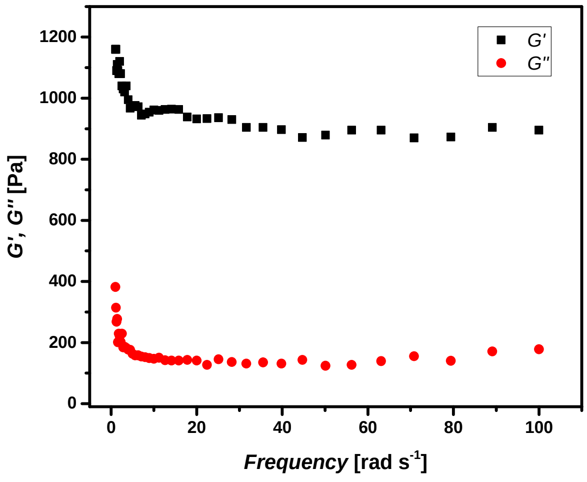

Figure S16. $POEGMA_{22k}$ 1.7 wt.%/α-CD 10.4 wt.%: Comparison of storage (*G'*, black) and loss modulus (*G''*, red) values against frequency.

Temperature treatment – temperature and time dependency

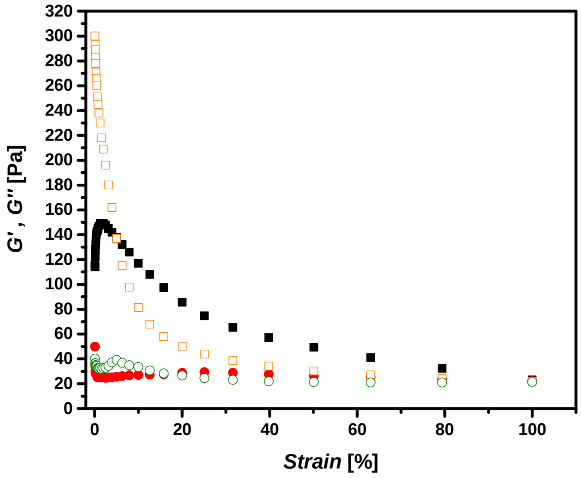

Figure S17. PVP_{41k}-*b*-$POEGMA_{22k}$ 3.3 wt.%/α-CD 10.4 wt.% after heating to 50 °C and cooling to ambient temperature): Comparison of storage (*G'*, black and orange) and loss modulus (*G''*, red and green) values against strain, back (open) and forth (filled) process.

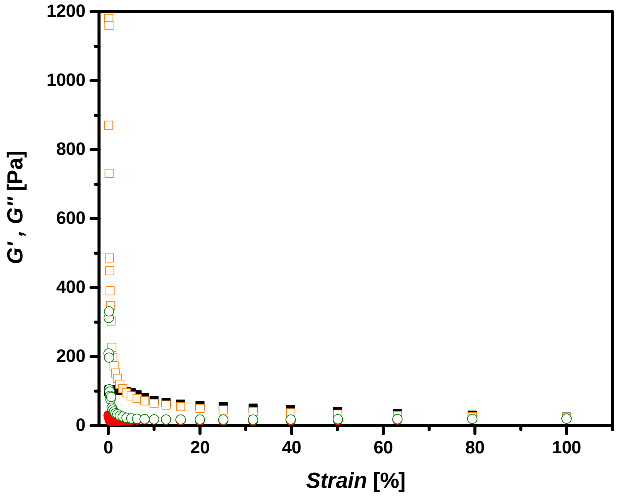

Figure S18. PVP$_{41k}$-*b*-POEGMA$_{22k}$ 3.3 wt.%/α-CD 10.4 wt.% after heating to 55 °C and cooling to ambient temperature): Comparison of storage (*G'*, black and orange) and loss modulus (*G''*, red and green) values against strain, back (open) and forth (filled) process.

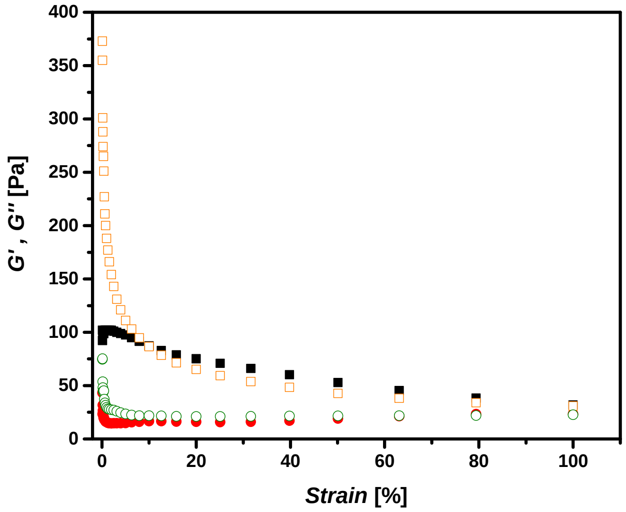

Figure S19. PVP$_{41k}$-*b*-POEGMA$_{22k}$ 3.3 wt.%/α-CD 10.4 wt.% after heating to 60 °C and cooling to ambient temperature): Comparison of storage (*G'*, black and orange) and loss modulus (*G''*, red and green) values against strain, back (open) and forth (filled) process.

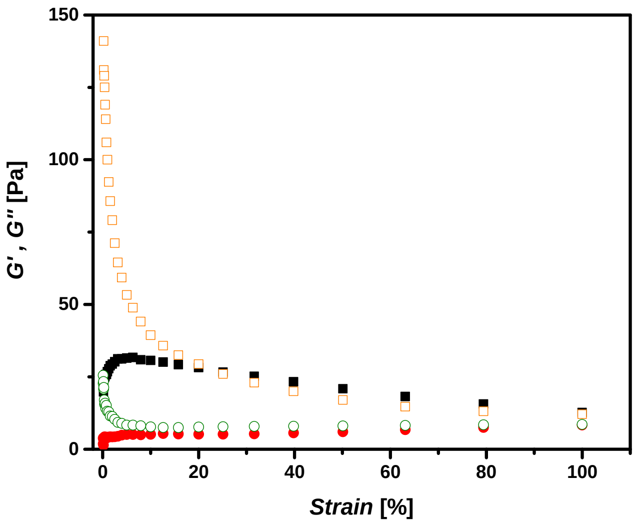

Figure S20. PVP$_{41k}$-*b*-POEGMA$_{22k}$ 3.3 wt.%/α-CD 10.4 wt.% after heating to 65 °C and cooling to ambient temperature for 1 hour): Comparison of storage (*G'*, black and orange) and loss modulus (*G''*, red and green) values against strain, back (open) and forth (filled) process.

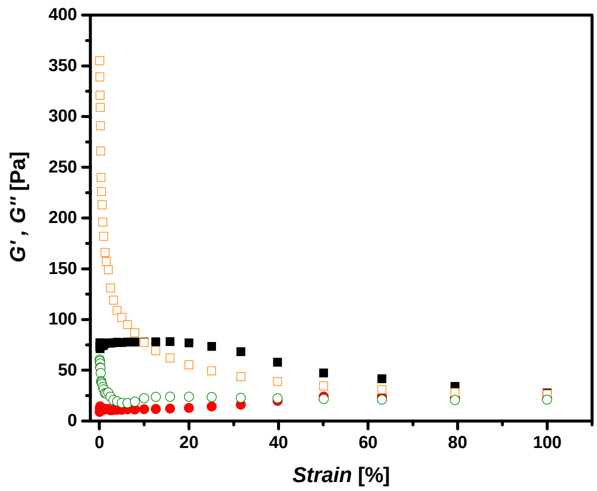

Figure S21. PVP$_{41k}$-*b*-POEGMA$_{22k}$ 3.3 wt.%/α-CD 10.4 wt.% after heating to 65 °C and cooling to ambient temperature for 6 hours): Comparison of storage (*G'*, black and orange) and loss modulus (*G''*, red and green) values against strain, back (open) and forth (filled) process.

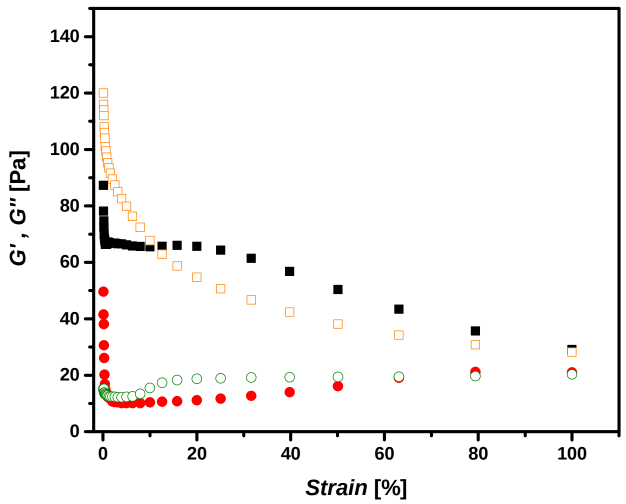

Figure S22. PVP$_{41k}$-*b*-POEGMA$_{22k}$ 3.3 wt.%/α-CD 10.4 wt.% after heating to 65 °C and cooling to ambient temperature for 16 hours): Comparison of storage (*G'*, black and orange) and loss modulus (*G''*, red and green) values against strain, back (open) and forth (filled) process.

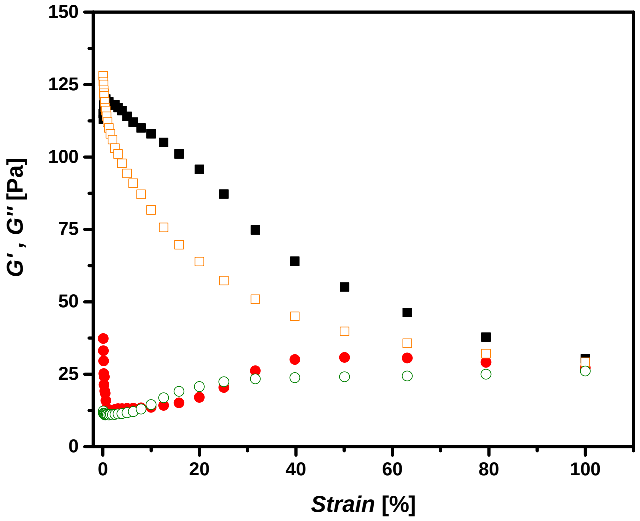

Figure S23. PVP$_{41k}$-*b*-POEGMA$_{22k}$ 3.3 wt.%/α-CD 10.4 wt.% after heating to 65 °C and cooling to ambient temperature for 48 hours): Comparison of storage (*G'*, black and orange) and loss modulus (*G''*, red and green) values against strain, back (open) and forth (filled) process.

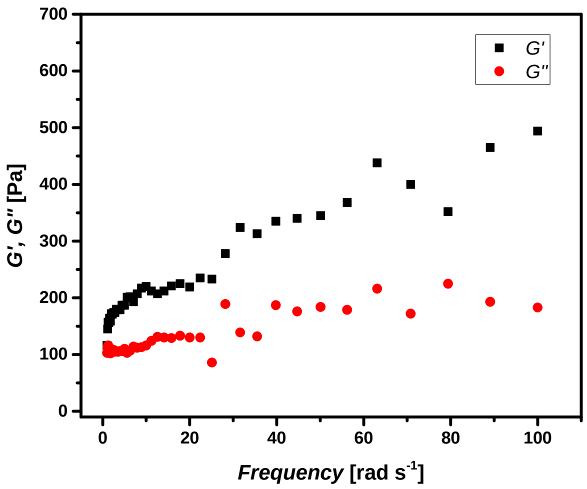

Figure S24. PVP$_{41k}$-*b*-POEGMA$_{22k}$ 3.3 wt.%/α-CD 10.4 wt.% after heating to 65 °C and cooling to ambient temperature): Comparison of storage (*G'*, black) and loss modulus (*G''*, red) values against frequency.

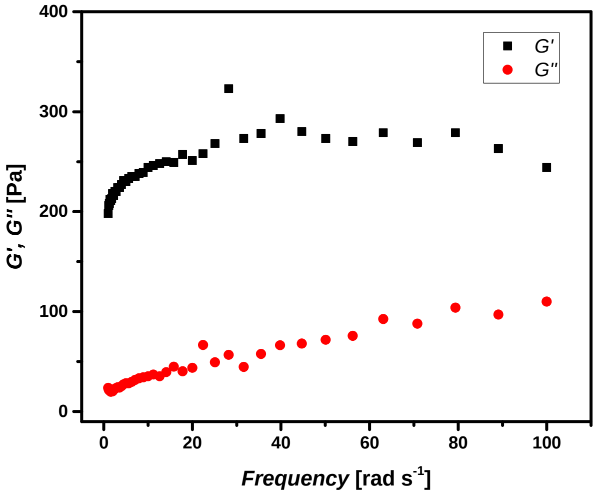

Figure S25. PVP$_{41k}$-*b*-POEGMA$_{22k}$ 3.3 wt.%/α-CD 10.4 wt.% after heating to 85 °C and cooling to ambient temperature): Comparison of storage (*G'*, black) and loss modulus (*G''*, red) values against frequency.

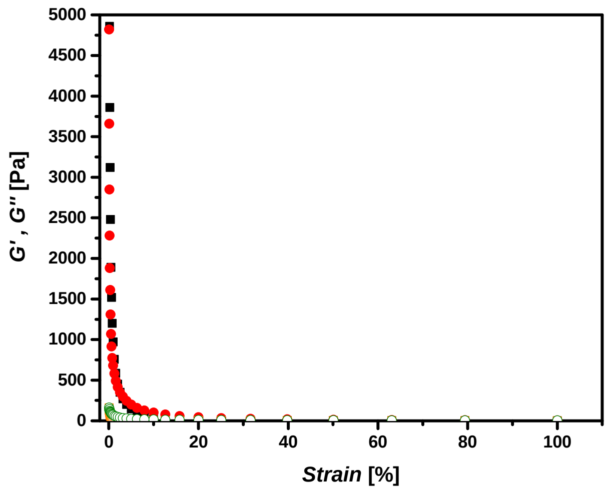

Figure S26. POEGMA$_{22k}$ 1.7 wt.%/α-CD 10.4 wt.% after heating to 85 °C and cooling to ambient temperature for 24 hours: Comparison of storage (*G'*, black and orange) and loss modulus (*G''*, red and green) values against strain, back (open) and forth (filled) process.

Cyclic heating

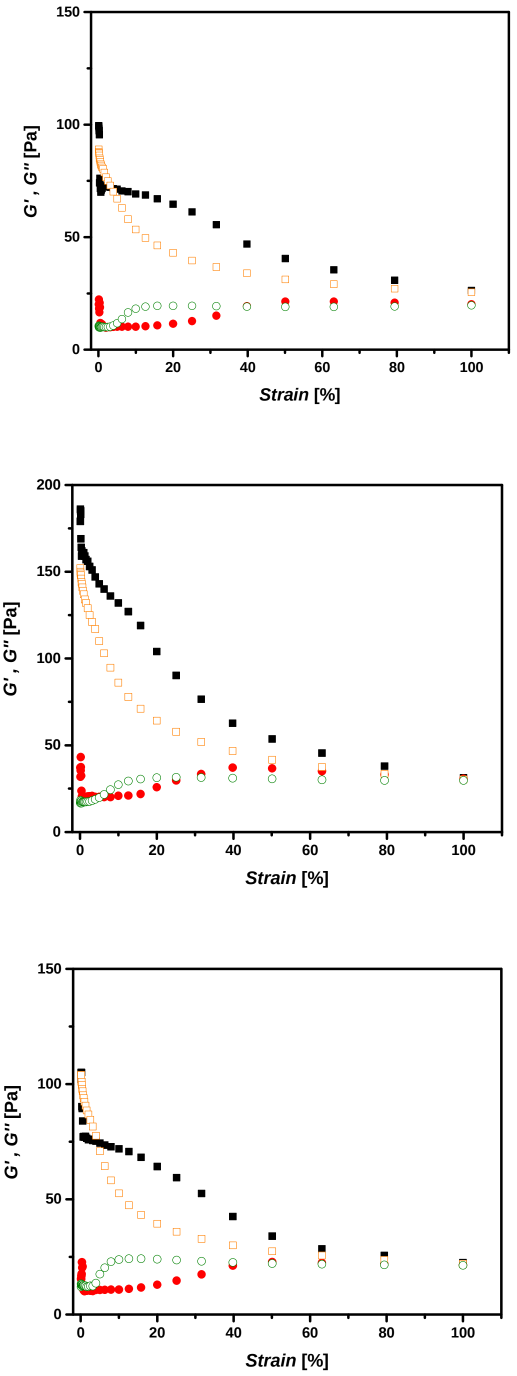

Figure S27. PVP$_{41k}$-*b*-POEGMA$_{22k}$ 3.3 wt.%/α-CD 10.4 wt.% after heating cycles (85 °C to ambient temperature): Comparison of storage (*G'*, black and orange) and loss modulus (*G''*, red

and green) values against strain, back (open) and forth (filled) process. Top: 2 cycles, middle: 4 cycles, bottom: 6 cycles.

Microstructure

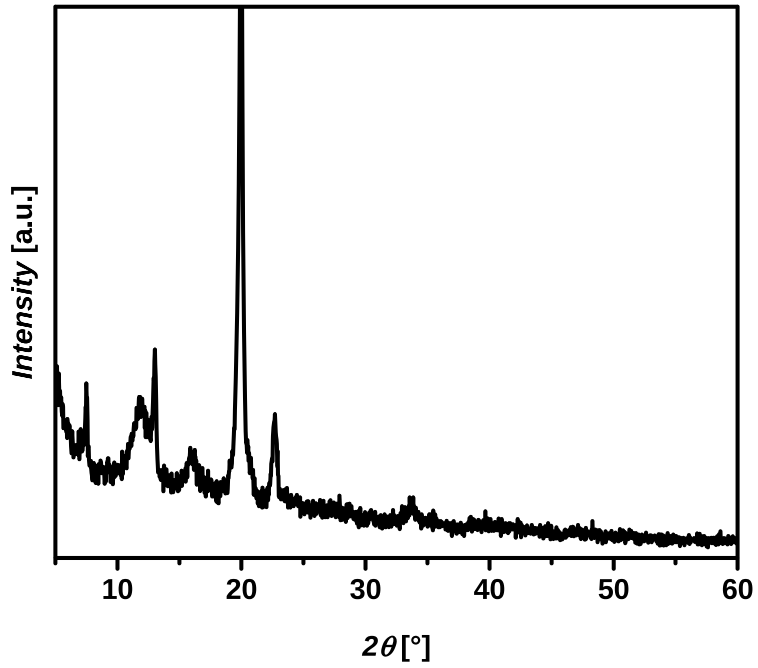

Figure S28. XRD pattern of $POEGMA_{22k}$ 1.7 wt.%/α-CD 10.4 wt.% hydrogel after freeze drying.

1. Quemener, D.; Davis, T. P.; Barner-Kowollik, C.; Stenzel, M. H., *Chem. Commun.* **2006,** (48), 5051-5053.
2. Al Nakeeb, N.; Willersinn, J.; Schmidt, B. V. K. J., *Biomacromolecules* **2017,** *18* (11), 3695-3705.
3. Liu, P.; Song, J., *J. Polym. Sci., Part A: Polym. Chem.* **2016,** *54* (9), 1268-1277.
4. Willersinn, J.; Bogomolova, A.; Brunet Cabré, M.; Schmidt, B. V. K. J., *Polym. Chem.* **2017,** *8*, 1244-1254.
